# RExPRT: a machine learning tool to predict pathogenicity of tandem repeat loci

**DOI:** 10.1186/s13059-024-03171-4

**Published:** 2024-01-31

**Authors:** Sarah Fazal, Matt C. Danzi, Isaac Xu, Shilpa Nadimpalli Kobren, Shamil Sunyaev, Chloe Reuter, Shruti Marwaha, Matthew Wheeler, Egor Dolzhenko, Francesca Lucas, Stefan Wuchty, Mustafa Tekin, Stephan Züchner, Vanessa Aguiar-Pulido

**Affiliations:** 1 Dr. John T. Macdonald Foundation Department of Human Genetics and John P. Hussman Institute for Human Genetics, University of Miami Miller School of Medicine, Biomedical Research Building (BRB), Miami, FL 33136 USA; 2 Department of Biomedical Informatics, Harvard Medical School, Boston, MA 02155 USA; 3 Stanford Center for Undiagnosed Diseases, Stanford University, Stanford, CA 94305 USA; 4 Division of Cardiovascular Medicine, Department of Medicine, Stanford University School of Medicine, Stanford, CA USA; 5 Illumina Inc., San Diego, CA 92112 USA; 6 Department of Computer Science, Delft University of Technology, Delft, The Netherlands; 7 Department of Computer Science, University of Miami, Miami, FL USA; 8 Deptartment of Biology, University of Miami, Miami, FL USA; 9 Sylvester Comprehensive Cancer Center, University of Miami Miller School of Medicine, Miami, FL USA

**Keywords:** Repeat expansions, Machine learning, Rare diseases

## Abstract

Expansions of tandem repeats (TRs) cause approximately 60 monogenic diseases. We expect that the discovery of additional pathogenic repeat expansions will narrow the diagnostic gap in many diseases. A growing number of TR expansions are being identified, and interpreting them is a challenge. We present RExPRT (Repeat EXpansion Pathogenicity pRediction Tool), a machine learning tool for distinguishing pathogenic from benign TR expansions. Our results demonstrate that an ensemble approach classifies TRs with an average precision of 93% and recall of 83%. RExPRT’s high precision will be valuable in large-scale discovery studies, which require prioritization of candidate loci for follow-up studies.

**Supplementary Information:**

The online version contains supplementary material available at 10.1186/s13059-024-03171-4.

## Background

Tandem repeats (TRs) are regions of the DNA that are composed of repeating motifs that vary between 2 and 6 base pairs (bp) in length [1]. There are 1.5 million TR loci scattered throughout the human genome [2]. Expansions of TRs can produce changes in the underlying genetic architecture, thus impacting molecular processes through the RNA or protein level [3]. Currently, only ~ 50 known disease-causing tandem repeat expansion loci have been identified, [3] a small minority compared to the 4482 genes associated with Mendelian diseases (OMIM) [4]. We speculate that there are many more disease-associated TR loci to be discovered, and progress has been hindered by the technical challenges associated with identifying long expanded repeats from short-read sequencing data. Specifically, correctly aligning short reads containing fully repetitive sequence and determining repeat lengths based on incomplete sequence information has posed significant complications. However, with the advancement of tools such as ExpansionHunter [5, 6] and GangSTR [7], as well as the emergence of databases characterizing tandem repeats (TRs) in control samples [8], the identification of rare repeat expansions from patient genomes is now possible. Previously, we showed that genomes from healthy individuals each have an average of ~ 250 common large TRs (> 175 bp) and a median of three rare large TRs [8]. This indicates that rare repeat expansions can be benign and population allele frequency alone cannot reliably distinguish pathogenic loci.

To assess the pathogenicity of single-nucleotide variants (SNVs) and small indels, detailed guidelines assist with filtering variants that go beyond consideration of allele frequencies [9, 10]. Tools such as ANNOVAR [11] and Ensembl’s Variant Effect Predictor (VEP) [12] annotate variants with their functional impact on the corresponding protein to assist with such filtering. The commonly used pathogenicity predictor Combined Annotation-Dependent Depletion (CADD) scores simple variants based on sequence context including evolutionary constraint, epigenetics, and gene model annotations [13]. Numerous additional tools are available for variant annotation, prediction, and prioritization in SNVs and small indels.

For structural variants (SVs), several tools have recently been developed to aid in variant prioritization. Specifically focusing on structural variants in exons, StrVCTVRE uses supervised learning to distinguish pathogenic from benign SVs, accounting for features such as conservation, expression, and exon structure [14]. Another tool, SVpath, predicts the pathogenicity of exonic SVs by incorporating features that are based on functional impact scores of overlapping SNVs, as well as gene level and transcriptomics scores [15]. Developing this approach further, DeepSVP integrates ranking of noncoding variants and incorporates phenotype information using a deep learning approach to improve the selection of patient-specific variants in a more precise manner [16].

In the field of TRs, efforts to prioritize variants have focused on an underlying assumption that TR constraint correlates with pathogenicity. Gymrek et al. showed this to be true for select early-onset disease loci such as RUNX2 and HOXD13 [17]. However, according to their methodology for predicting mutational constraint, late-onset disease loci such as ATXN7 are not highly constrained. Since many repeat expansion diseases manifest later in life, mutational constraint alone is an unreliable prioritization metric. One of the few existing tools for TR prioritization—SISTR—is based on a population genetics framework [18]. It calculates a selection coefficient, which incorporates measures of mutation, genetic drift, and negative natural selection. While alleles that are negatively selected are predicted to be more deleterious, these values correlate with mutational constraint and are therefore subject to the same limitations. Additionally, SISTR was demonstrated for use in a complex genetic disorder, autism, whose underlying genetic etiology differs from those of rare Mendelian diseases [18].

**Fig. 1:**
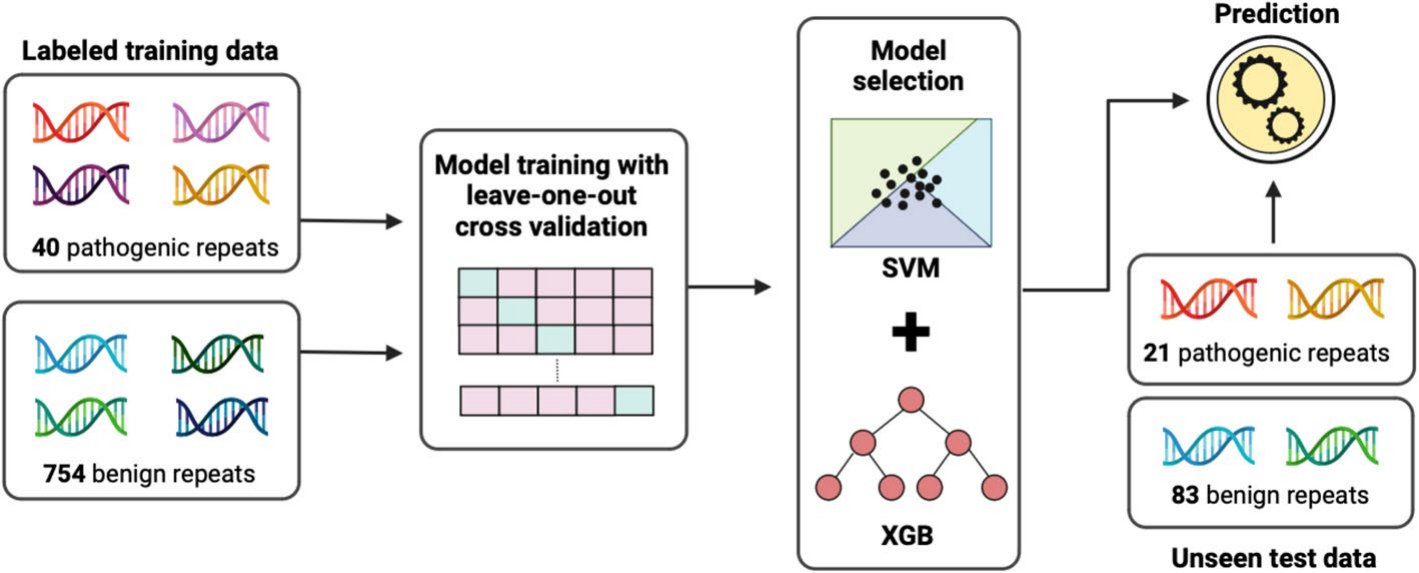
Methodology of RExPRT. RExPRT was trained on 40 known pathogenic TRs and 745 benign TRs that are commonly expanded in the 1000 Genomes Project controls. These TRs were annotated with features, which are used in a supervised statistical learning approach to classify TRs as pathogenic or benign. Seven different models were trained and validated using the LOOCV technique. Two models were selected and fine-tuned to create an optimized ensemble method for ranking repeats. Twenty one pathogenic TRs and 83 rare, benign TRs were used for testing RExPRT’s performance

Currently, there are no prioritization models built on labelled training data containing examples of both pathogenic and benign TRs. The challenge lies especially in the limited training data, considering there are only ~ 50 known pathogenic repeat expansion loci discovered to date [3]. To address this gap, we present RExPRT, a supervised machine learning-based *R*epeat *Ex*pansion *P*athogenicity p*R*ediction *T*ool. RExPRT is the first tool applicable for both early- and late-onset TR-driven rare monogenic diseases that can score and categorize TR loci as pathogenic and benign (Fig. 1).

## Results

### Pathogenic TRs are enriched in regulatory regions and in specific areas within genes

**Fig. 2:**
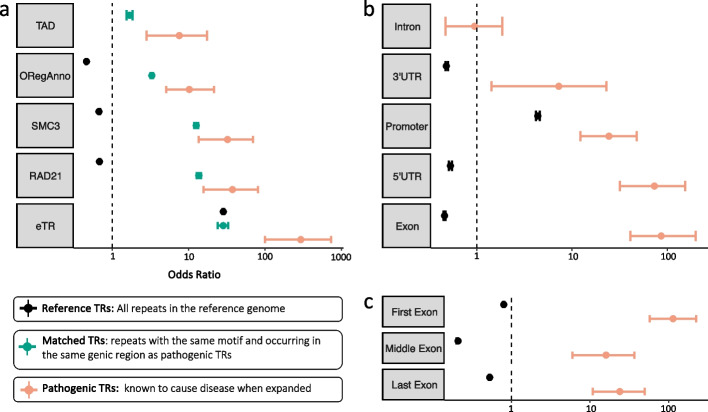
Genomic regions with enrichment of pathogenic TRs. **a** Odds ratios from Fisher’s exact tests for pathogenic TRs, matched TRs, and reference TRs in their intersection with TAD boundaries, open regulatory regions, SMC3 and RAD21 transcription factor binding sites, and eTRs. **b** Odds ratios from Fisher’s exact tests for pathogenic TRs and reference TRs in their intersection with different genic regions. **c** Odds ratios from Fisher’s exact tests for pathogenic TRs and reference TRs in their intersection with different exonic regions

We investigated whether known pathogenic TRs are enriched in any particular regions of the genome compared with matched TRs and all reference TRs. We found that pathogenic TRs are enriched in topologically associated domain (TAD) boundaries (odds ratio (OR) = 7.55; 95% confidence interval (CI) = [2.82, 17.37]; *p* = 9.66e^−05^), open regulatory regions (ORegAnno) (OR = 10.21; 95% CI = [5.09, 21.56]; *p* = 1.70e^−12^), as well as SMC3 (OR = 32.34; 95% CI = [13.54, 69.56]; *p* = 7.46e^−11^) and RAD21 transcription factor binding sites (OR = 37.51; 95% CI = [15.71, 80.70]; *p* = 2.06e^−11^) (Fig. 2a). Furthermore, pathogenic TRs are also more likely to be classified as expression TRs (eTRs) (OR = 294.52, 95% CI = [99.81, 732.38]; *p* = 1.94e^−13^), which are repeats whose length has been reported to be directly associated with transcript levels of the respective gene [2].

Pathogenic TRs are also enriched in 3′UTRs (OR = 7.27; 95% CI [1.43, 22.97]; *p* = 9.79e^−03^), promoters (OR = 24.41; 95% CI = [12.24, 47.76]; *p* = 4.25e^−16^), 5′UTRs (OR = 73.20; 95% CI = [31.90, 153.71]; *p* = 2.75e^−15^), and exons (OR = 86.15; 95% CI = [41.10, 197.73]; *p* < 2.2e^−16^) while we did not find any significant enrichment in introns (OR = 0.95; 95% CI = [0.47, 1.86]; *p* = 1) (Fig. 2b). Upon further exploration, we found that there is significantly more enrichment of pathogenic TRs in the first exon of genes (OR = 113.60; 95% CI = [57.47, 223.43]; *p* < 2.2e^−16^) compared to the middle and last exons (Fig. 2c).

### RExPRT achieves 99% accuracy using LOOCV

**Fig. 3:**
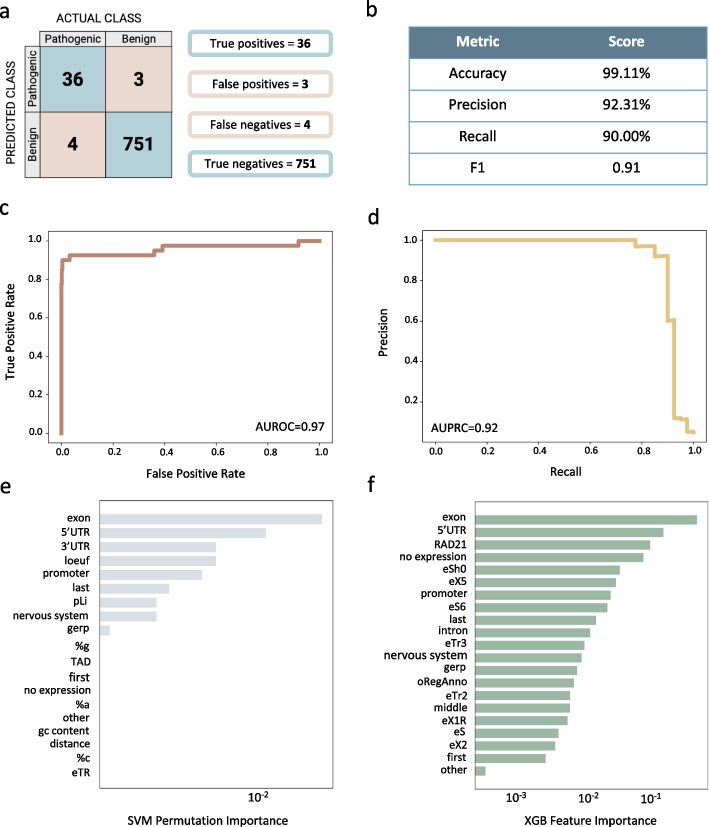
Results from ensemble of SVM and XGB with LOOCV on training dataset. **a** Confusion matrix outlining the number of true positives, false positives, false negatives, and true negatives on the training set. **b** Calculations of the accuracy, precision, recall, and F1 scores. **c** The ROC curve for the ensemble model and its AUC value. **d** PRC curve for the ensemble model and its AUC value. **e** Permutation importance, indicating features that allow decision-making in the SVM model and **f** the XGB model

RExPRT is an ensemble method combining the two best performing models based on the LOOCV results: SVM and XGB. It excels in its low false positive rate (0.38%) while still attaining an excellent recall of 90% (Fig. 3a, b). This means we have a good balance between precision (92.31%) and recall, resulting in an F1 score of 0.91 (Fig. 3b). The corresponding ROC curve in Fig. 3c highlights RExPRT’s prowess as evidenced by a high AUROC value of 0.97. While ROC curves are a standard way to present machine learning results, a PRC is more informative with an imbalanced dataset. The PRC curve is also robust (AUPRC = 0.92) and illustrates the fine balance between our precision and recall rates (Fig. 3d). Since pathogenic TRs compose [5%] of the dataset, an AUPRC value of [0.05] represents random guessing performance. Therefore, RExPRT’s performance is considerably better than random guessing. Figure 3 e and f represent the results of feature or permutation importance analyses for each of the two models. We find that both models are extremely reliant on whether the TR is in an exon or 5′UTR. The SVM model utilizes pLi and LOEUF scores, and percentage calculations of nucleotides within the TR motifs. On the other hand, XGB applies the S2SNet topological indices calculations for its predictions (Fig. 3e, f). The two models complement one another, as together they boost the overall performance of RExPRT.

RExPRT misclassifies only four pathogenic TRs as benign: RFC1, FXN, CNBP, and ATXN10. Interestingly, all four of these TRs are in intronic regions within their respective genes. None of them overlap TAD boundaries, RAD21 binding sites, or open regulatory regions and are not characterized as eTRs. They are also not in 3′UTRs or 5′UTRs, nor in promoters except for CNBP. Their GERP scores are extremely close to 0 (range = 0–0.039), but the average GERP score for TRs classified as true positives is 0.67. The average GERP score for true negative TRs is − 0.10, and there is a statistically significant difference between the two groups (*p* = 2.80e − 09) (Additional file 1: Fig. S1). The motifs for each of these TRs are unique within the group of pathogenic TRs in the training dataset but are found in the benign group. All these characteristics could explain why these four pathogenic TRs are misclassified as benign.

### RExPRT maintains a low false positive rate on the testing dataset

**Fig. 4:**
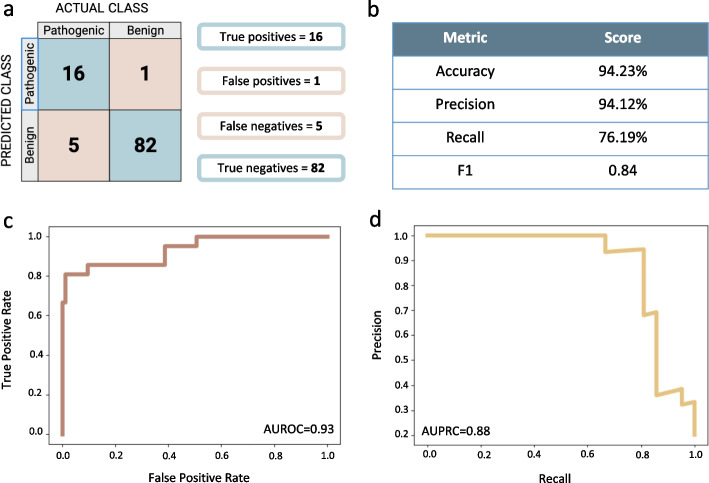
Performance metrics for RExPRT on the testing dataset. **a** The confusion matrix results from running RExPRT on the testing dataset of 93 TRs. **b** Calculations of the accuracy, precision, recall, and F1 scores. **c** ROC curve and its AUC value. **d** PRC curve and its AUC value

Next, we ran RExPRT on our testing dataset to assess its performance. The unseen data was comprised of 21 additional pathogenic TRs and 83 rare, benign TR expansions. We found that RExPRT maintained a low false positive rate (1.20%), while its recall reduced to 76.19% (Fig. 4a, b). Since our precision (94.12%) is better than our recall here, the F1 score drops down to 0.84. The ROC curve maintains a steep slope, but its AUC value drops to 0.93 (Fig. 4c). The PRC demonstrates high performance despite the imbalanced dataset with an AUC value of 0.88 (Fig. 4d).

There are five pathogenic TRs in the testing dataset whose motifs are a variation of CGG. Since there are 12 such pathogenic TRs in the training dataset, there are ample examples for RExPRT to learn their correct classification. Indeed, RExPRT classified all five of these TRs with CGG motifs as pathogenic. An additional five pathogenic TRs are all intronic TTTCA expansions in different genes that cause various forms of familial adult myoclonic epilepsy (FAME). There are only two such examples in the training dataset, SAMD12 and DAB1, both of which are correctly classified by RExPRT during LOOCV. In the case of the five FAME loci in the testing dataset, YEATS2 is the only one that is correctly classified as pathogenic, but the XGB score for STARD7 is extremely close to the threshold for pathogenicity with a probability of 0.49. Importantly, these loci are intronic and follow similar patterns in their characteristics as the pathogenic TRs in the training dataset that failed to be classified correctly. Finally, FGF14 is a newly discovered locus that is also intronic. It is unique in that it is commonly observed at sizes above 175 bp, and therefore could resemble some of the common benign TRs in the training dataset.

### Further validation by random splitting of dataset

To further demonstrate the sufficiency of our data and the reliability of our results, we conducted additional analyses. From our full dataset, we generated five separate training and testing datasets by random division. Application of the ensemble model resulted in an average precision of 89% and average recall of 85% (Additional file 1: Table S3). The corresponding mean values for the initial train/test split are 93 and 83%, extremely similar to those from the random split. This highlights the maintenance of robustness and high performance of our models across different circumstances.

### RExPRT is able to correctly classify TR loci associated with late-onset disorders

To compare RExPRT’s performance on early-onset and late-onset disorders, we created a scatterplot showing the correlation between the age of onset for repeat expansion diseases, and the RExPRT pathogenicity score for the associated TR locus (Additional file 1: Fig. S2). This analysis revealed a week negative correlation between these two variables, with Spearman’s rho value of − 0.35. Notably, we observe that many late-onset disorders are correctly classified as pathogenic by RExPRT.

### RExPRT identifies ~ 30,000 TRs in the reference genome that may be pathogenic if expanded

**Fig. 5:**
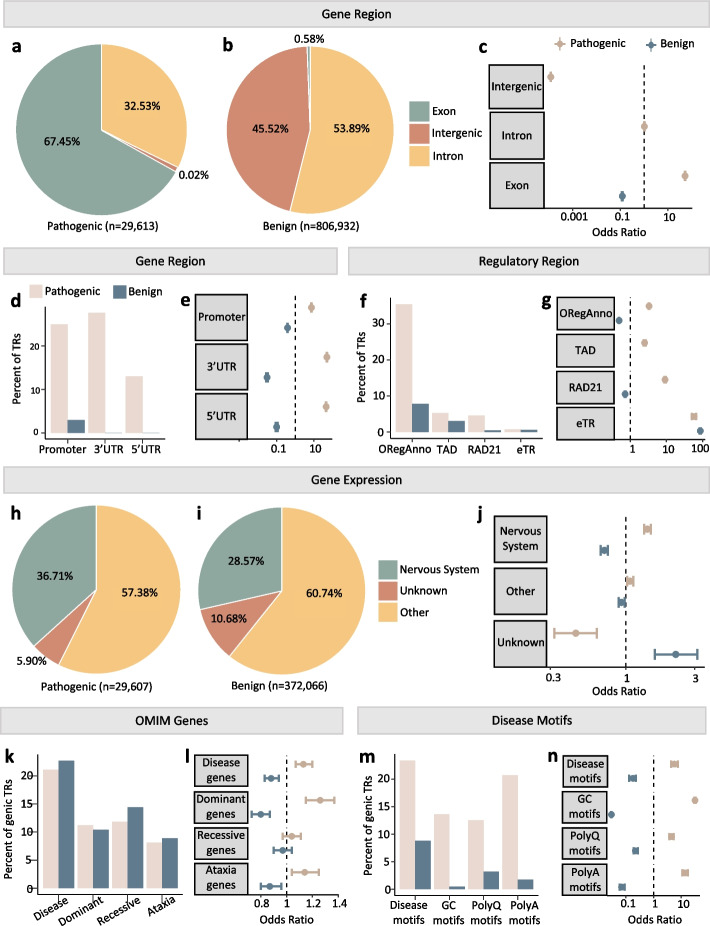
Characterization of ~ 800,000 reference TRs analyzed by RExPRT. **a** TRs in the reference genome that are predicted pathogenic, and their distribution among exonic, intronic, and intergenic categories. **b** TRs in the reference genome that are predicted benign, and their distribution among exonic, intronic, and intergenic categories. **c** Odds ratios for pathogenic and benign TRs in their intersection with exonic, intronic, and intergenic regions of the genome, notably showing an enrichment of pathogenic TRs in exons. **d** Distribution of pathogenic and benign TRs in promoters, 3′UTRs, and 5′UTRs. **e** Odds ratios for pathogenic and benign TRs demonstrating an enrichment of pathogenic TRs in promoters, 3′UTRs, and 5′UTRs. **f** Distribution of pathogenic and benign TRs in open regulatory regions, TAD boundaries, RAD21 transcription factor binding sites, and as eTRs. **g** Odds ratios for pathogenic and benign TRs demonstrating an enrichment of pathogenic TRs in open regulatory regions, TAD boundaries, RAD21 transcription factor binding sites, and eTRs. **h** TRs in genic regions that are predicted to be pathogenic, and their tissue expression. **i** TRs in genic regions that are predicted to be benign, and their tissue expression. **j** Odds ratios for pathogenic and benign TRs in their tissue distributions demonstrating an enrichment for pathogenic TRs in nervous system tissues. **k** Distribution of pathogenic and benign TRs in OMIM disease genes, dominant disease genes, recessive disease genes, and ataxia genes. **l** Odds ratios for pathogenic and benign TRs demonstrating an enrichment of pathogenic TRs in OMIM disease genes, dominant genes, and ataxia genes. **m** Distribution of pathogenic and benign TRs with known repeat expansion disorder disease motifs, pure GC motifs, polyglutamine (polyQ) motifs, and polyalanine (polyA) motifs. **n** Odds ratios for pathogenic and benign TRs demonstrating an enrichment of pathogenic TRs with known repeat expansion disease motifs, pure GC motifs, polyQ motifs, and polyA motifs

We ran RExPRT on ~ 800,000 TRs with motif lengths between 3 and 8 bp listed in the hg19 reference genome. This resulted in 29,613 TRs classified as pathogenic if expanded, 67.45% of which are exonic, and 32.53% are intronic (Fig. 5a). Of the benign category, only 0.58% are exonic, and 53.89% are intronic, with the remaining being intergenic (Fig. 5b). Fisher’s exact tests demonstrate a clear enrichment for pathogenic TRs in exons (OR = 53.13; 95% CI = [51.78, 54.36]; *p* < 2.2e^−16^) (Fig. 5c). Such an observation is not surprising, since expansions in coding regions could alter the structure of the protein. More than half of the TRs predicted as pathogenic are in promoters (24.95%), 3′UTRs (27.56%), or 5′UTRs (13.02%), while only ~ 3% of the benign TRs fall within these regions (Fig. 5d). Fisher’s exact tests confirm an enrichment for pathogenic TRs in promoters (OR = 7.33; 95% CI = [7.13, 7.54]; *p* < 2.2e^−16^), 3′UTRs (OR = 49.11; 95% CI = [47.65, 50.62]; *p* < 2.2e^−16^), and 5′UTRs (OR = 45.08; 95% CI = [43.56, 46.74]; *p* < 2.2e^−16^) (Fig. 5e).

We find a greater number of pathogenic TRs overlapping open regulatory regions (35.47%), TAD boundaries (5.22%), RAD21 transcription factor binding sites (4.59%), and being classified as eTRs (0.76%), compared with benign TRs (Fig. 5f). Fisher’s exact tests demonstrate enrichment of pathogenic TRs in TAD boundaries (OR = 2.50; 95% CI = [2.34, 2.66]; *p* < 2.2e^−16^) and RAD21 binding sites (OR = 9.16; 95% CI [8.71, 9.63]; *p* < 2.2e^−16^), and in open regulatory regions (OR = 3.30; 95% CI = [3.22, 3.38]; *p* < 2.2e^−16^). Expression TRs are found in both groups, since these are a subset of the larger group of reference repeats (Fig. 5g).

Next, we removed all intergenic TRs from our pathogenic and benign groups and investigated gene expression. We found that 36.71% of pathogenic TRs are expressed in nervous system tissues, with 57.38% being expressed in other tissues (Fig. 5h). In the benign category, 28.57% are expressed in nervous system tissues, and 60.74% in other tissues (Fig. 5i). Fisher’s exact tests demonstrated an enrichment of pathogenic TRs in nervous system tissues (OR = 1.41; 95% CI = [1.34, 1.49]; *p* < 2.2e^−16^) (Fig. 5j).

Pathogenic TRs occur at similar rates compared to benign TRs in OMIM disease genes (21.08%), dominant disease genes (11.20%), recessive disease genes (11.84%), and ataxia genes (8.14%) (Fig. 5k). However, Fisher’s exact tests demonstrate a slight enrichment of pathogenic TRs in OMIM disease genes (OR = 1.13; 95% CI = [1.07, 1.20]; *p* = 3.20e^−05^), as well as in dominant genes specifically (OR = 1.26; 95% CI = [1.15, 1.37]; *p* = 1.10e^−07^) and ataxia genes (OR = 1.14; 95% CI = [1.04, 1.25]; *p* = 3.76e^−03^), but not in recessive genes (OR = 1.04; 95% CI = [0.97, 1.11]; *p* = 0.32) (Fig. 5l).

Upon exploring the motifs for TRs in each group, we found that pathogenic TRs have a greater percentage of known repeat expansion disease motifs (23.37%), GC motifs (13.61%), polyQ motifs (12.52%), and polyA motifs (20.71%) compared to benign TRs (Fig. 5m). Fisher’s exact tests demonstrated that pathogenic TRs are enriched in disease motifs (OR = 5.45; 95% CI = [4.12, 7.522), pure GC motifs (OR = 32.56; 95% CI = [31.08, 34.10]; *p* < 2.2e^−16^), polyQ motifs (OR = 4.35; 95% CI = [3.63, 5.24]; *p* < 2.2e^−16^), and polyA motifs (OR = 14.83; 95% CI = [11.73, 18.99]; *p* < 2.2e^−16^) (Fig. 5n).

### RExPRT identifies several interesting candidate genes in the Undiagnosed Diseases Network (UDN) cohort data

We processed 2982 genomes from the UDN through the outlier pipeline described in “Materials and methods” that detects rare, repeat expansions in each case. This resulted in 449 candidate TRs, or rare, genic TRs that originate from a reference repeat locus. Of these, 23 genes had TRs that were predicted as pathogenic by RExPRT. Ten of these are already known pathogenic sites, which leaves a total of 13 novel strong candidates for further investigation. More specifically, only three of these are observed in multiple affected patients in a heterozygous state; FAM193B, FRA10AC1, and CLEC2B.

Interestingly, the FAM193B candidate was found in two affected siblings, both with oculopharyngodistal myopathy (OPDM). Notably, this disorder has already been linked to expansions in four other genes: LRP12 [19], GIPC1 [20], NOTCH2NLC [21], and RILPL1 [22]. All these expansions are composed of a CGG motif and occur in the 5′UTR of their respective gene. The same pattern is observed with the TR we found in FAM193B, making it a strong novel candidate variant for the phenotype. The heterozygous expansion was confirmed in the affected siblings using Oxford Nanopore long-read sequencing, but we are lacking confirmation in additional families. Further investigation into the pathogenicity and mechanism of disease of the expanded TR is underway.

The FRA10AC1 locus has been previously described as likely benign for a phenotype of intellectual disability. The authors concluded that the expansion may be pathogenic only in a homozygous state since methylation of the CGG repeat was observed in a single affected patient and carrier [23]. However, it may be premature to draw this conclusion since specific repeat sizes were not measured, nor were methylation levels assessed in a cohort of individuals.

The CLEC2B candidate TR was observed in two patients with unsteady gait. The first patient has been partially diagnosed with hearing loss, but this does not explain their symptoms of hypotonia, seizures, and unsteady gait. The second patient has muscle weakness and atrophy, as well as unsteady gait.

## Discussion

We built RExPRT to address the lack of available tools for assessing the pathogenicity of repeats. Our previous data demonstrated that rare repeat expansions are often found in healthy controls [8], which indicates that simply selecting TR expansions by allele frequency is not sufficient to determine pathogenicity. RExPRT incorporates information on the genetic architecture of a TR locus, such as its proximity to regulatory regions, TAD boundaries, and evolutionary constraints. It further includes information on gene expression and the DNA motif a TR is composed of. These features enable RExPRT to predict TR pathogenicity with an accuracy of 96.67%. RExPRT excels at having a robust precision due to its low false positive rate. However, its limitations lie in its recall which averages at 83.10%. In the field of Mendelian discovery genetics, the optimization of precision is preferable to avoid a long list of candidates with many false positive TR expansions that require costly, time-consuming, and labor-intensive experimental validation.

Nonetheless, future versions of RExPRT should focus on improving the recall of TRs that are intronic, require motif changes, and are recessive. A major limitation comes from the general paucity of pathogenic TRs to train on, particularly TRs that are recessive, intronic, and require motif changes. Statistical learning approaches like the one employed in this study are inherently dependant on existing knowledge and identification of patterns that align with known data. In the future, by providing the machine learning algorithms with a more comprehensive set of examples to learn from, its performance will likely increase. Additionally, the incorporation of features that are more informative for these problematic TRs could be considered. Finally, since different loci will have different size thresholds for pathogenicity, integration of more accurate genotyping data from long-read sequencing technology may enable future versions of RExPRT to suggest minimum thresholds.

Applying RExPRT to ~ 800,000 reference TR loci, we found that ~ 30,000 were classified as pathogenic. It is important to note here that realistically, many of these TRs will never be observed as expanded because they are in fact stable for molecular reasons or affect essential genes or genomic regions that are embryonic lethal. Our results demonstrate that pathogenic TRs, according to RExPRT, are enriched in exons, promoters, 3′UTRs, 5′UTRs, TAD boundaries, RAD21 binding sites, eTRs, and genes expressed in the nervous system. Pathogenic TRs are also enriched in disease genes, particularly dominantly inherited genes, and ataxia genes. It is important to note that many repeat expansion disorders are multifaceted in their mechanism of action, and often involve both gain and loss of function effects. Considering many repeat expansion diseases present with ataxia as a primary phenotype, the enrichment in ataxia genes is a particularly interesting observation. Not surprisingly, our predicted pathogenic TRs are enriched in known disease motifs, including polyQ and polyA. If these TRs are found to be expanded in patients with phenotypes similar to known diseases with the same motifs, they would be extremely strong candidates. FAM193B is one such example; we found the CGG expansion in a family with OPMD, a disease already associated with four other CGG expansions in different genes [19–22]. Pure GC-rich motifs seem to be particularly enriched in the pathogenic group, even in the training and testing datasets. These regions may be associated with mechanisms that make them pathogenic upon expansion. While we identified several compelling candidate repeat expansions in the UDN cohort, it is essential to highlight that the mean age of onset in the UDN is 11 years ± 18. It is therefore plausible that other cohorts specifically focused on late-onset disorders could uncover additional expansions associated with such conditions.

The limitations of our study are primarily attributed to our utilization of a small and imbalanced dataset, a common challenge encountered in the realm of biological research. Specifically, our set of known pathogenic expansions was limited to a total of 62 TRs. Moreover, we were compelled to rely on certain assumptions pertaining to our benign TRs datasets. These assumptions were based on the premise that frequently occurring large TRs are likely benign and that rare expansions observed in individuals diagnosed with Mendelian disorders can also be considered benign. Furthermore, since these TRs were selected from short-read sequencing data, which may not accurately genotype large repeats, a TR was considered large or expanded if its length exceeded the read length (> 175 bp). To mitigate the impact of these limitations, our research methodology incorporated several measures. We employed a leave-one-out cross validation approach and used a diverse array of performance metrics to comprehensively evaluate the model’s effectiveness. Importantly, we opted against downsampling our already limited dataset or artificially augmenting it by generating synthetic datapoints. This decision was made to preserve the integrity of this foundational study and prevent the introduction of artificial elements.

## Conclusion

RExPRT has established the possibility of using machine learning to classify repeat expansion variation. Our work lays the groundwork for similar tools that can be built in the future, mitigating some of its limitations.

## Materials and methods

### Identification of significant data features

To select features that may be relevant for classifying TR loci as pathogenic or benign, we downloaded 30 different annotation datasets from a variety of sources including UCSC’s Table Browser, PsychENCODE, and relevant publications (Additional file 1: Table S1). We converted all dataset files into BED format. First, we created a list of 40 known pathogenic TRs which are listed as represented in the training dataset in Table 1. These TRs were included in initial analyses because they are well established in their causal link to disease [24–26]. Next, we obtained a dataset of all TRs cataloged in the reference genome from UCSC’s Table Browser (Simple Repeats table in hg19). Finally, we used this reference TR dataset to create a set of “matched TRs,” which included all TRs that have a disease motif and occur in the same genic region. For example, all TRs with a CAG motif that occur in exons would be part of the matched TR dataset.

**Table 1:** Pathogenic TRs used in training/LOOCV and testing datasets. RExPRT ensemble classification, as well as both SVM and XGB scores are provided for each pathogenic TR. Additionally, information is provided for each locus including associated disease, motif, the gene region in which the TR is located, age-of-onset, inheritance pattern of the associated disease, whether a motif change is required to pathogenic, and whether the motif was uniquely represented among other pathogenic TRs in the training dataset

Gene	Disease	Age of onset	Motif	Gene region	Inheritance	Motif change	Unique motif	SVM score	XGB score	RExPRT classification	Dataset
AFF2	Fragile X syndrome, FRAXE type	2–10 years	CCG	5′UTR	X-Linked	No	No	0.99	0.91	Pathogenic	Training
AFF3	Intellectual disability associated with fragile site FRA2A	0–7 years	CGG	Intron	Dominant	No	No	0.98	0.64	Pathogenic	Testing
AR	Spinal and bulbar muscular atrophy, Kennedy disease	20–49 years	CAG	Exon	X-Linked	No	No	0.97	1.00	Pathogenic	Training
ARX	Early infantile epileptic encephalopathy	< 1 year	GCG	Exon	X-Linked	No	No	0.99	0.97	Pathogenic	Training
ARX	Partington syndrome	1–3 years	GCN	Exon	X-Linked	No	No	0.98	0.99	Pathogenic	Testing
ATN1	Dentatorubral-pallidoluysian atrophy	1–72 years	CAG	Exon	Dominant	No	No	0.97	0.99	Pathogenic	Training
ATXN1	Spinocerebellar ataxia type 1	13–60 years	CAG	Exon	Dominant	No	No	0.97	0.99	Pathogenic	Training
ATXN10	Spinocerebellar ataxia type 10	12–48 years	ATTCT	Intron	Dominant	No	Yes	0.00	0.00	Benign	Training
ATXN2	Spinocerebellar ataxia type 2	25–50 years	CAG	Exon	Dominant	No	No	0.87	1.00	Pathogenic	Training
ATXN3	Spinocerebellar ataxia type 3, Machado-Joseph disease	10–50 years	CAG	Exon	Dominant	No	No	0.63	1.00	Pathogenic	Training
ATXN7	Spinocerebellar ataxia type 7	0–50 years	CAG	Exon	Dominant	No	No	1.00	1.00	Pathogenic	Training
ATXN8OS	Spinocerebellar ataxia type 8	1–73 years	CTG	3′UTR	Dominant	No	No	0.99	0.90	Pathogenic	Training
BEAN1	Spinocerebellar ataxia type 31	20–72 years	TGGAA	Intron	Dominant	Yes	Yes	0.51	0.04	Pathogenic	Training
C9orf72	Amyotrophic lateral sclerosis, frontotemporal dementia	27–85 years	GGGGCC	Intron	Dominant	No	Yes	0.90	0.94	Pathogenic	Training
CACNA1A	Spinocerebellar ataxia type 6	19–73 years	CAG	Exon	Dominant	No	No	0.92	1.00	Pathogenic	Training
CBL2	Jacobsen syndrome, fragile site FRAX11B	< 1 year	CGG	5′UTR	Dominant	No	No	0.98	0.98	Pathogenic	Testing
CNBP	Myotonic dystrophy type 2	30–40 years	CCTG	Intron	Dominant	No	Yes	0.07	0.00	Benign	Training
COMP	Multiple epiphyseal dysplasia	13 years	GTC	Exon	Dominant	No	Yes	0.96	1.00	Pathogenic	Testing
CSTB	Myoclonic epilepsy of Unverricht and Lundborg	6–15 years	CCCCGCCCCGCG	5′UTR	Recessive	No	Yes	0.83	0.52	Pathogenic	Training
DAB1	Spinocerebellar ataxia type 37	18–64 years	TTTCA	Intron	Dominant	Yes	No	0.00	0.53	Pathogenic	Training
DIP2B	Intellectual developmental disorder, FRA12A type	1 year	CGG	5′UTR	X-Linked	No	No	0.97	1.00	Pathogenic	Testing
DMPK	Myotonic dystrophy type 1	0–70 years	CTG	3′UTR	Dominant	No	No	0.76	0.98	Pathogenic	Training
FGF14	Spinocerebellar ataxia 27B	46–77 years	GAA	Intron	Dominant	No	No	0.02	0.00	Benign	Testing
FMR1	Fragile X syndrome	2–65 years	CGG	5′UTR	X-Linked	No	No	0.97	0.99	Pathogenic	Training
FOXL2	Blepharophimosis, epicanthus inversus, and pitosis	< 1 year	GCC	Exon	Dominant	No	No	0.99	0.99	Pathogenic	Training
FXN	Friedrich ataxia	5–25 years	GAA	Intron	Recessive	No	Yes	0.00	0.01	Benign	Training
GIPC1	Oculopharyngodistal myopathy	10–29 years	CGG	5′UTR	Dominant	No	No	0.95	0.99	Pathogenic	Training
GLS	Glutaminase deficiency	0–1 years	GCA	5′UTR	Recessive	No	No	0.93	1.00	Pathogenic	Training
HOXA13	Hand-foot-genital syndrome 1	< 1 year	GCN	Exon	Dominant	No	No	0.97	1.0	Pathogenic	Testing
HOXA13	Hand-foot-genital syndrome 2	< 1 year	GCG	Exon	Dominant	No	No	1.00	0.99	Pathogenic	Training
HOXA13	Hand-foot-genital syndrome 3	< 1 year	GCN	Exon	Dominant	No	No	1.00	0.99	Pathogenic	Testing
HOXD13	Syndactyly	< 1 year	GCG	Exon	Dominant	No	No	0.83	1.00	Pathogenic	Training
HTT	Huntington disease	10–70 years	CAG	Exon	Dominant	No	No	0.97	1.00	Pathogenic	Training
JPH3	Huntington disease-like 2	12–66 years	CTG	3′UTR	Dominant	No	No	0.56	0.62	Pathogenic	Training
LRP12	Oculopharyngodistal myopathy type 1	7–50 years	CGG	5′UTR	Dominant	No	No	0.98	0.99	Pathogenic	Training
MARCHF6	Familial adult myoclonic epilepsy type 3	10–40 years	TTTCA	Intron	Dominant	Yes	No	0.00	0.01	Benign	Testing
NIPA1	Amyotrophic lateral sclerosis	19–66 years	GCG	Exon	Dominant	No	No	0.98	1.0	Pathogenic	Testing
NOP56	Spinocerebellar ataxia type 36	48–57 years	GGCCTG	Intron	Dominant	No	Yes	0.67	0.66	Pathogenic	Training
NOTCH2NLC	Neuronal intranuclear inclusion disease	16–76 years	GGC	5′UTR	Dominant	No	No	0.88	0.71	Pathogenic	Training
NUTMB-AS1	Oculopharyngeal myopathy with leukoencephalopathy 1	15–40 years	GGC	Exon	Dominant	No	No	0.83	0.04	Pathogenic	Testing
PABPN1	Oculopharyngeal muscular dystrophy	26–65 years	GCG	Exon	Dominant	No	No	0.90	0.80	Pathogenic	Training
PHOX2B	Congenital central hypoventilation syndrome	0–20 years	GCN	Exon	Dominant	No	No	0.71	0.99	Pathogenic	Training
PPP2R2B	Spinocerebellar ataxia type 12	8–55 years	CAG	5′UTR	Dominant	No	No	0.87	1.00	Pathogenic	Training
PRDM12	Hereditary sensory and autonomic neuropathy type VIII	< 1 year	GCC	Exon	Recessive	No	No	0.98	0.98	Pathogenic	Testing
RAPGEF2	Familial adult myoclonic epilepsy type 7	18–37 years	TTTCA	Intron	Dominant	Yes	No	0.02	0.03	Benign	Testing
RFC1	Cerebellar ataxia, neuropathy, and vestibular areflexia syndrome	33–71 years	AAGGG	Intron	Recessive	Yes	Yes	0.01	0.00	Benign	Training
RILPL1	Oculopharyngodistal myopathy type 4	14–27 years	CGG	5′UTR	Dominant	No	No	0.48	0.72	Pathogenic	Testing
RUNX2	Cleidocranial dysplasia	< 1 year	GCN	Exon	Dominant	No	No	1.00	0.99	Pathogenic	Training
SAMD12	Familial adult myoclonic epilepsy type 1	18–50 years	TTTCA	Intron	Dominant	Yes	No	0.94	0.00	Pathogenic	Training
SOX3	X-linked mental retardation	0–9 years	GCC	Exon	X-Linked	No	No	0.95	0.98	Pathogenic	Training
STARD7	Familial adult myoclonic epilepsy type 2	5–60 years	TTTCA	Intron	Dominant	Yes	No	0.05	0.49	Benign	Testing
TBP	Spinocerebellar ataxia type 17	3–55 years	CAG	Exon	Dominant	No	No	0.98	1.00	Pathogenic	Training
TBX1	Tetralogy of Fallot	< 1 year	GCN	Exon	Dominant	No	No	0.96	0.99	Pathogenic	Testing
TCF4	Fuchs endothelial corneal dystrophy 3	32–70 years	CTG	Intron	Dominant	No	No	0.62	0.96	Pathogenic	Training
THAP11	Spinocerebellar ataxia	4–51 years	CAG	Exon	Dominant	No	No	0.96	0.99	Pathogenic	Testing
TNRC6A	Familial adult myoclonic epilepsy type 6	20–70 years	TTTCA	Intron	Dominant	Yes	No	0.00	0.02	Benign	Testing
XYLT1	Baratela-Scott syndrome	< 1 year	GGC	5′UTR	Recessive	No	No	0.99	0.04	Pathogenic	Training
YEATS2	Familial adult myoclonic epilepsy type 4	10–33 years	TTTCA	Intron	Dominant	Yes	No	0.02	0.63	Pathogenic	Testing
ZIC2	Holoprosencephaly-5	< 1 year	GCN	Exon	Dominant	No	No	0.98	1.00	Pathogenic	Training
ZIC3	X-linked VACTERL syndrome	< 1 year	GCN	Exon	X-Linked	No	No	0.97	0.98	Pathogenic	Testing
ZNF713	Autism spectrum disorder associated with fragile site FRA7A	Early	CGG	5′UTR	Dominant	No	No	0.97	0.99	Pathogenic	Testing

The three TR datasets (pathogenic TRs, reference TRs, and matched TRs) were then compared individually with each of the 30 annotation files. When evaluating a feature, we employed bedtools fisher, which takes two sets of genomic intervals as input (e.g., pathogenic TRs and ORegAnno regions) and performs Fisher’s exact test, evaluating the amount of overlap between the two sets of intervals. This test is designed to determine whether there is a statistically significant nonrandom association between these two sets of genomic intervals. For the example provided, the presence of a significant *p*-value in Fisher’s exact test would indicate that the intervals in the pathogenic TRs are enriched in ORegAnno regions. It highlights that pathogenic TRs are more likely to occur in ORegAnno regions than expected by chance. Using bedtools fisher, we generated contingency tables for all pairs of comparisons, then calculated 95% confidence intervals for the odds ratios using fisher.test() from the R stats package. If the 95% confidence interval of the odds ratio for an annotation’s intersection with pathogenic TRs was higher than the 95% confidence interval for the matched TRs, the annotation feature was considered significantly enriched for pathogenic TRs. For genic annotation features (3′UTR, 5′UTR, exon, intron, and promoter), as presented in Fig. 2b and c, the comparison was made between pathogenic TRs and reference TRs instead to avoid bias, since matched TRs already selected for certain genic regions.

### Quantitative and multivalued features

We further selected additional annotations to test in our models (Additional file 1: Table S2). These included datasets with numerical values rather than the purely categorical datasets discussed above. For the features described above, TRs were given a value of 1 if the locus intersected with the annotation feature, and a value of 0 if there was no intersection. Quantitative datasets that we incorporated include pLI scores, LOEUF scores [27], and GERP scores [28]. The basis for including these is due to their significance in assessing pathogenicity of SNVs. We also created a feature that calculated the distance between the TR locus and the nearest gene, which was important for TRs that are intergenic.

Additionally, we incorporated information regarding the motif assuming a motif-phenotype correlation exists as proposed by Ishiura et al., where TRs with the same motifs, occurring in the same genic regions—albeit in different genes entirely—can produce the same phenotypes, suggesting TR motif may be important in its pathogenicity [29]. We calculated the percentages of each nucleotide in the motif and provided a GC content as well. For motif analysis, we also used the Sequence to Star Network (S2SNet) approach [30], which can transform any character-based sequence into a graph-based star-shaped complex network. We characterized the star network’s topological indices (Tis) with calculations of different metrics, including its Shannon entropy, spectral moments, Harray number, Wiener index, Gutman topological index, Schultz topological index, Balaban distance connectivity index, Kier-Hall connectivity index, and Randic connectivity index [30]. S2SNet was downloaded from GitHub and run using the default parameters on the command line. For the input, we created a sequence of 10 repeating units of the TR motif.

Finally, we accounted for tissue expression of the genes that harbored TRs. We created a categorical feature reflecting the tissue where each gene had its maximum expression according to data from the GTEx Portal (date of accession: September 2021). The TR was then assigned to one of three possible categories: expressed in neurological tissue, expressed in another, or unknown expression.

### Training and testing dataset creation and preprocessing

To train RExPRT, we used the same 40 known pathogenic loci that were assessed with Fisher’s exact tests above. Based on our previous work, [8] we used a set of 754 TR loci that were commonly expanded in the 1000 Genomes Project controls as negative training data. Specifically, we selected loci that were present at a size of > 175 bp in more than 1% of the control samples. Since these TRs are commonly expanded, we presumed that they are benign. To annotate our TRs with the features discussed above, we created an input file containing the reference coordinates of the locus in hg19 (chromosome, start position, and end position), as well as the motif. After annotation with the features described, we used the OneHotEncoder from Scikit-Learn to preprocess the data. This was used for categorical variables including GTEx, gene region (intron/exon/intergenic), and gene location (first/middle/last).

For the testing dataset, we used the 21 remaining pathogenic TR loci listed in Table 1. These were loci that were either discovered more recently, have only been found in a small number of affected families, or were previously missed [3]. For the negative loci in the test set, we used sites that can be presumed benign, but are still rare since RExPRT will have to distinguish between rare benign TRs and rare pathogenic TRs. We decided to use 83 rare TRs that were candidate TRs resulting from running the outlier pipeline described subsequently on our 102 positive controls. Since these genomes correspond to patients that were all diagnosed with a Mendelian repeat expansion disorder (caused by a single gene), other TR expansions in these genomes are presumed to be benign. Therefore, we combined all the candidate TRs from these genomes and excluded all known pathogenic TRs to produce a list of 83 rare, benign TRs.

### Model testing and assessment

We tested seven different statistical learning models: logistic regression, *k*-nearest neighbors (KNN), support vector machine (SVM), random forest (RF), decision tree (DT), gradient boosted decision tree (XGB), and linear discriminant analysis (LDA). To evaluate the performance of these models, we used a leave-one-out cross validation (LOOCV) approach. The LOOCV approach is useful here because we have a small positive training dataset of known pathogenic TRs. For models that are sensitive to scaling such as SVM, we used StandardScaler to standardize features by subtracting the mean and scaling by the standard deviation. To assess model performance, we obtained the confusion matrix and determined overall accuracy, precision, recall, and F1 metrics. We also produced receiver operating characteristic curve (ROC) and precision-recall curves (PRC) and calculated the area under the curve for each one. All coding was done in Python using Scikit-Learn. Formulas for calculations are listed below:

1 $$Accuracy=\frac{true\;positives\;\left(tp\right)+true\;negatives\;(tn)}{tp+tn+false\;positives\;\left(fp\right)+false\;negatives\;(fn)}\times100$$

2 $$Precision=\frac{tp}{tp+fp}$$

3 $$Recall= \frac{tp}{tp+fn}$$

4 $$F1=\frac{2 \times Precision \times Recall}{Precision+Recall}$$

### Feature selection

To further improve the accuracy of the two best performing models, we plotted a feature importance graph (XGB) and a permutation importance graph (SVM) to visualize the significance of each feature to the models. We then removed features that were ranked low on the list, indicating that the feature was not adding significant value in the decision-making process. We only removed features if they did not impact confusion matrix scores or the overall area under the ROC curve (AUROC) score.

Additionally, we created a correlation plot of all features and removed features that were highly correlated. In particular, correlated features that produced the highest AUROC value if retained were kept, ensuring that removal of features did not reduce the AUROC score.

### Hyperparameter tuning

We used Scikit-Learn’s GridSearchCV to fine-tune the SVM and XGB models. For SVM, we tuned three parameters: C, gamma, and kernel. For XGB, we tuned two parameters: number of estimators, and max depth.

### Ensemble method

To improve the overall recall of RExPRT, we devised an ensemble approach, which encompasses the two different models described above (SVM and XGB) [31]. The classification of the TR by the ensemble model will be pathogenic if either of the models predict likelihood of pathogenicity. The threshold is set to a standard 0.5 probability, above which the TR is classified as pathogenic. Furthermore, RExPRT also provides a confidence score, which is the sum of the SVM and XGB scores. Figure 1 is a schematic illustrating the complete methodology and workflow of RExPRT, covering training, LOOCV, model selection, and predictions on the testing dataset.

### Random splitting of data

We combined our initial training and testing datasets, then generated five separate training and testing datasets by random division (2/3 for training and 1/3 for testing). This was done using scikit-learn’s train_test_split() function. We then employed the ensemble model and generated summary metrics for these datasets.

### Age of onset analysis

We calculated the midpoint of the observed age of onset ranges for each disorder. Next, we generated a scatter plot where each point corresponds to a disease-associated pathogenic TR locus. The *x*-axis represents the midpoint of the observed range of onset ages for the associated disease (refer to Table 1), while the *y*-axis reflects the RExPRT ensemble score. Subsequently, we calculated Spearman’s rank correlation coefficient (rho) for these two variables.

### Analysis of ~ 800,000 reference repeats classified by RExPRT

We applied RExPRT to *836,545* TRs with motif lengths between 3 and 8 bp listed in the hg19 reference genome. We separated TRs into those classified as pathogenic by RExPRT and those classified as benign. We then characterized the genomic features of pathogenic TRs and benign TRs. For each feature, we created two plots:

- Bar graph or pie chart: this represents the fraction of pathogenic or benign TRs that overlap a particular feature relative to the total number of TRs within the group.
- Odds ratio graph: this graph evaluates whether the observed percentage above is significantly deviated from what would be expected by random chance, considering the coverage of that feature within the entire genome.

Bedtools fisher was used to perform Fisher’s exact tests and calculate odds ratios for gene regions and regulatory regions as described above. For gene expression characterization, we excluded intergenic TRs. We then performed Fisher’s exact tests in R, first creating a contingency table for benign and pathogenic TRs. To do this, we calculated the number of genes that overlapped in the different sets of tissues with the two groups of classified TRs.

For analysis of Online Mendelian Inheritance in Man (OMIM) genes, we downloaded the list of genes from their website, and filtered for those that are Mendelian. For those genes that are dominant and recessive, we used this same dataset and filtered for the respective subtype. The ataxia gene list was obtained from GeneDx’s Ataxia Xpanded panel (https://www.genedx.com/tests/detail/ataxia-xpanded-panel-887). The odds ratios were obtained by calculating Fisher’s exact tests in R, as described for the gene expression data.

To analyze the disease motifs, we filtered all pathogenic and benign TRs for those which contained a known repeat expansion disease motif (Table 1). We included each window shift of the motif as well as its reverse complement. For pure GC motifs, we filtered for TRs that only contained G and C in their motif. For polyglutamine (polyQ) and polyalanine (polyA) motifs, we began with only coding TRs in each group and filtered for the trinucleotides which code for these amino acids. Since we do not have the coding frame information, many of these will not actually code for glutamine or alanine, so this is an overestimation. Odds ratios were calculated in R, as described for the gene expression, and disease gene categories.

### Undiagnosed Diseases Network (UDN) sample processing

Patient fastq files were aligned with Burrows–Wheeler aligner (BWA) to the GRCh38 reference genome [32]. The resulting SAM files were converted to BAM files. Duplicates were removed with Picard tools MarkDuplicates—https://github.com/broadinstitute/picard. After sorting and reindexing, base quality score recalibration (BQSR) was performed using genome analysis toolkit (GATK) [33]. Next, ExpansionHunter Denovo (EHDn) was run on the resulting BAM files, using additional parameters “–min-unit-len 3 –max-unit-len 8” [6]. The outputs of EHDn for each case was then aggregated separately to the outputs for the 2405 samples from the 1000 Genomes Project controls using EHDn’s helper scripts to allow depth normalization. Finally, the bed file output was expanded from its sparse encoding into a dense matrix format using R. This dense matrix of depth-normalized anchored in-repeat read (IRR) counts was used as the input for the outlier pipeline.

### Outlier pipeline

After sample processing, each case is represented by a dense matrix of TRs with anchored IRR counts for the case as well as all controls. Summary statistics were calculated for all TRs before filtering. For each TR, the outlier pipeline calculates a *z*-score, a kernel density estimation, and the percentage of controls with anchored IRR counts above that seen for the patient sample. The *z*-score for a case is a measure of their anchored IRR count in terms of the number of standard deviations above or below the mean anchored IRR counts observed in controls. The kernel density estimation gives the expected proportion of controls with anchored IRR counts above that of the patient, based on the density curve distribution of controls. All three measures give us an indication of the likelihood of the anchored IRR count for the patient sample being part of the distribution of controls. Downstream filtering selects for TRs with high *z*-scores, and low values for the other two measures. Specifically, the pipeline filters out TRs based on anchored IRR counts in patients (≥ 5), frequency of controls with at least 1 allele over 175 bp (< 1%), genomic region the TR is located in (≠ intergenic), and whether the TR stems from a reference repeat locus or *Alu* element. Moreover, we also filter out a list of 126 false positive sites that were selected based on their presence in a heterogenous group of ~ 600 disease genomes and low occurrence in the 1000 Genomes controls. It is thought that since these variants are too common in a cohort of rare disease cases, they cannot be causal variants. The final output is a list of candidate TRs for each patient genome. Therefore, here we define candidate TRs as TRs that are rare and expanded in a patient, occur within or close to genes, and stem from a reference repeat locus.

### Analysis of UDN genomes

We ran 2982 genomes (968 probands and affected/unaffected family members) from the UDN through our outlier pipeline, resulting in a list of candidate TRs that can be defined as rare, genic TR expansions that stem from a reference repeat locus. Since these TRs were called by ExpansionHunter Denovo (EHDn), they do not have precise locus specificity. EHDn provides coordinates of ~ 1–2000 bp surrounding the repeat. To run ExpansionHunter on these loci for determining repeat number and zygosity, we used ehdn-to-eh (https://github.com/francesca-lucas/ehdn-to-eh), which provides precise coordinates for the TR. These coordinates were then used to create the variant catalogs for running ExpansionHunter. Since the UDN genomes are aligned to hg38, we converted the loci into hg19 using UCSC’s LiftOver tool [34], and then ran RExPRT on the candidate TRs.

### Supplementary Information

**Additional file 1: Supplementary results.** Further details on features, age of onset analysis, and outcome metrics from randomly splitting the dataset into training and testing datasets multiple times [38, 39]. **Fig. S1.** GERP scores for TRs in the training dataset. **Fig. S2.** Age of onset for known repeat expansion disorders and the maximum RExPRT pathogenicity score predicted for the associated TR locus. **Table S1.** Features tested for significant association with pathogenic TRs. **Table S2.** Quantitative and multivalued features tested in machine learning models. **Table S3.** Summary metrics for randomly generated training and testing datasets.

**Additional file 2. ** Review history.

## Data Availability

The data used in this paper were sourced from our previous publication in *Scientific Data* [8], as well as those mentioned in Additional file 1: Table S1. Additionally, our 120 positive controls dataset (EGAD00001003562) is available through the European Genome-Phenome Archive  [35]. The Undiagnosed Diseases Network genomes are not publicly available and would require permissions for access. The main website of the UDN can be found at https://undiagnosed.hms.harvard.edu/about-us/. Please refer to the contact details on the webpage for instructions on obtaining data access as well as further details on genomes. The scripts to run RExPRT for any TR of interest, as well as the RExPRT scores for the reference TRs are available on Zenodo [36] as well as on GitHub at the following URL: https://github.com/ZuchnerLab/RExPRT  [37] RExPRT is provided under the terms and conditions of the MIT open-source license. We are also working towards implementation of RExPRT into GENESIS, a user-friendly point and click analysis tool.
