# Supplementary material for: RExPRT: a machine learning tool to predict pathogenicity of tandem repeat loci
Source: Genome Biol. 2024 Jan 31;25:39. doi: 10.1186/s13059-024-03171-4 (PMC10832122; doi:10.1186/s13059-024-03171-4)
Supplement: Supplementary file 1 — Additional file 1: Supplementary results. Further details on features, age of onset analysis, and outcome metrics from randomly splitting the dataset into training and testing datasets multiple times [38, 39]. Fig. S1. GERP scores for TRs in the training dataset. Fig. S2. Age of onset for known repeat expansion disorders and the maximum RExPRT pathogenicity score predicted for the associated TR locus. Table S1. Features tested for significant association with pathogenic TRs. Table S2. Quantitative and multivalued features tested in machine learning models. Table S3. Summary metrics for randomly generated training and testing datasets. [file 13059_2024_3171_MOESM1_ESM.docx]

Supplementary Materials

**Table S1: Features tested for significant association with pathogenic TRs.**

The source where the feature dataset was downloaded from is provided, as well as whether the feature demonstrated significance after conducting fisher’s exact tests.

| Feature | Data source | Significance |
| --- | --- | --- |
| 3'UTR | UCSC annotation | Significant |
| 5'UTR | UCSC annotation | Significant |
| Alu elements | HOMER annotation files | Nonsignificant |
| Cerebellum eQTL | UCSC table browser | Nonsignificant |
| Conserved distal enhancers | UCSC table browser | Nonsignificant |
| Constrained non-conserved regions (CNCRs) | Chen et al^38^ | Nonsignificant |
| CpG islands | UCSC table browser | Nonsignificant |
| CTCF (neural cells) | UCSC table browser | Nonsignificant |
| EP300 (neural cells) | UCSC table browser | Nonsignificant |
| eTR | Fotsing et al^2^ | Significant |
| Exon | UCSC annotation | Significant |
| FAIRE (frontal cortex) | UCSC table browser | Nonsignificant |
| H3K27ac (ES cells) | UCSC table browser | Nonsignificant |
| H3K4me3 (neurons) | UCSC table browser | Nonsignificant |
| Intron | UCSC annotation | Nonsignificant |
| LINE | HOMER annotation files | Nonsignificant |
| MXI1 (neural cells) | UCSC table browser | Nonsignificant |
| ncRNA | HOMER annotation files | Nonsignificant |
| Non constrained non-conserved regions (NCNCRs) | Chen et al^38^ | Nonsignificant |
| ORegAnno | UCSC table browser | Significant |
| Promoter | UCSC annotation | Significant |
| Pseudogene | HOMER annotation files | Nonsignificant |
| RAD21 (neural cells) | UCSC table browser | Significant |
| SINE | HOMER annotation files | Nonsignificant |
| SMC3 (neural cells) | UCSC table browser | Significant |
| TAD boundaries (cortical cells) | Sun et al^39^ | Nonsignificant |
| TAD boundaries (DLPFC cells) | PsychENCODE | Significant |
| TAD boundaries (ES cells) | Sun et al^39^ | Nonsignificant |
| TAD boundaries with high CpG (DLPFC cells) | PsychENCODE/UCSC table browser | Nonsignificant |
| TAD boundaries with low CpG (DLPFC cells) | PsychENCODE/UCSC table browser | Nonsignificant |

**Table S2: Quantitative and multivalued features tested in machine learning models.**

The source where the feature dataset was downloaded from is provided.

| Feature | Data source |
| --- | --- |
| pLI scores | gnomAD |
| LOEUF scores | gnomAD |
| GERP scores | Cooper et al^28^ |
| Distance to the nearest gene | UCSC annotation / Bedtools closest |
| Gene expression | GTEx |
| GC content of motif (%) | N/A |
| Percent of motif composed of A/T/C/G | N/A |
| S2Snet motif characteristics | Munteanu et al^30^ |

**Table S3: Summary metrics for randomly generated training and testing datasets.**

Table presents metrics for contingency table, accuracy, precision, recall, auPRC, and F1 score for both training and testing datasets. These datasets were generated by randomly dividing the complete dataset into a 2/3 training set and 1/3 testing set. This random split was performed five times, and the results for the ensemble model are provided.

| TRAIN | True positives | True negatives | False positives | False negatives | Accuracy | Precision | Recall | auPRC | F1 score |
| --- | --- | --- | --- | --- | --- | --- | --- | --- | --- |
| #1 | 31 | 550 | 7 | 10 | 0.97 | 0.82 | 0.76 | 0.84 | 0.78 |
| #2 | 36 | 552 | 5 | 5 | 0.98 | 0.88 | 0.88 | 091 | 0.87 |
| #3 | 33 | 552 | 5 | 8 | 0.98 | 0.87 | 0.80 | 0.83 | 0.84 |
| #4 | 35 | 554 | 3 | 6 | 0.98 | 0.92 | 0.85 | 0.86 | 0.89 |
| #5 | 32 | 552 | 5 | 9 | 0.98 | 0.86 | 0.78 | 0.86 | 0.82 |
| Average |  |  |  |  | **0.98** | **0.87** | **0.81** | **0.86** | **0.84** |

| TEST | True positives | True negatives | False positives | False negatives | Accuracy | Precision | Recall | auPRC | F1 score |
| --- | --- | --- | --- | --- | --- | --- | --- | --- | --- |
| #1 | 18 | 278 | 2 | 2 | 0.99 | 0.90 | 0.90 | 0.94 | 0.90 |
| #2 | 17 | 278 | 2 | 3 | 0.98 | 0.89 | 0.85 | 0.90 | 0.87 |
| #3 | 15 | 280 | 0 | 5 | 0.98 | 1.0 | 0.75 | 0.92 | 0.86 |
| #4 | 19 | 276 | 4 | 1 | 0.98 | 0.83 | 0.95 | 0.95 | 0.89 |
| #5 | 19 | 279 | 1 | 1 | 0.99 | 0.95 | 0.95 | 0.96 | 0.95 |
| Average |  |  |  |  | **0.99** | **0.91** | **0.88** | **0.93** | **0.89** |

**Fig. S1: GERP scores for TRs in the training dataset.**

GERP scores are plotted for TRs classified as “true positives” or “true negatives” by the RExPRT ensemble method. Wilcoxon signed-rank test p-value demonstrates a statistically significant difference between the two groups.


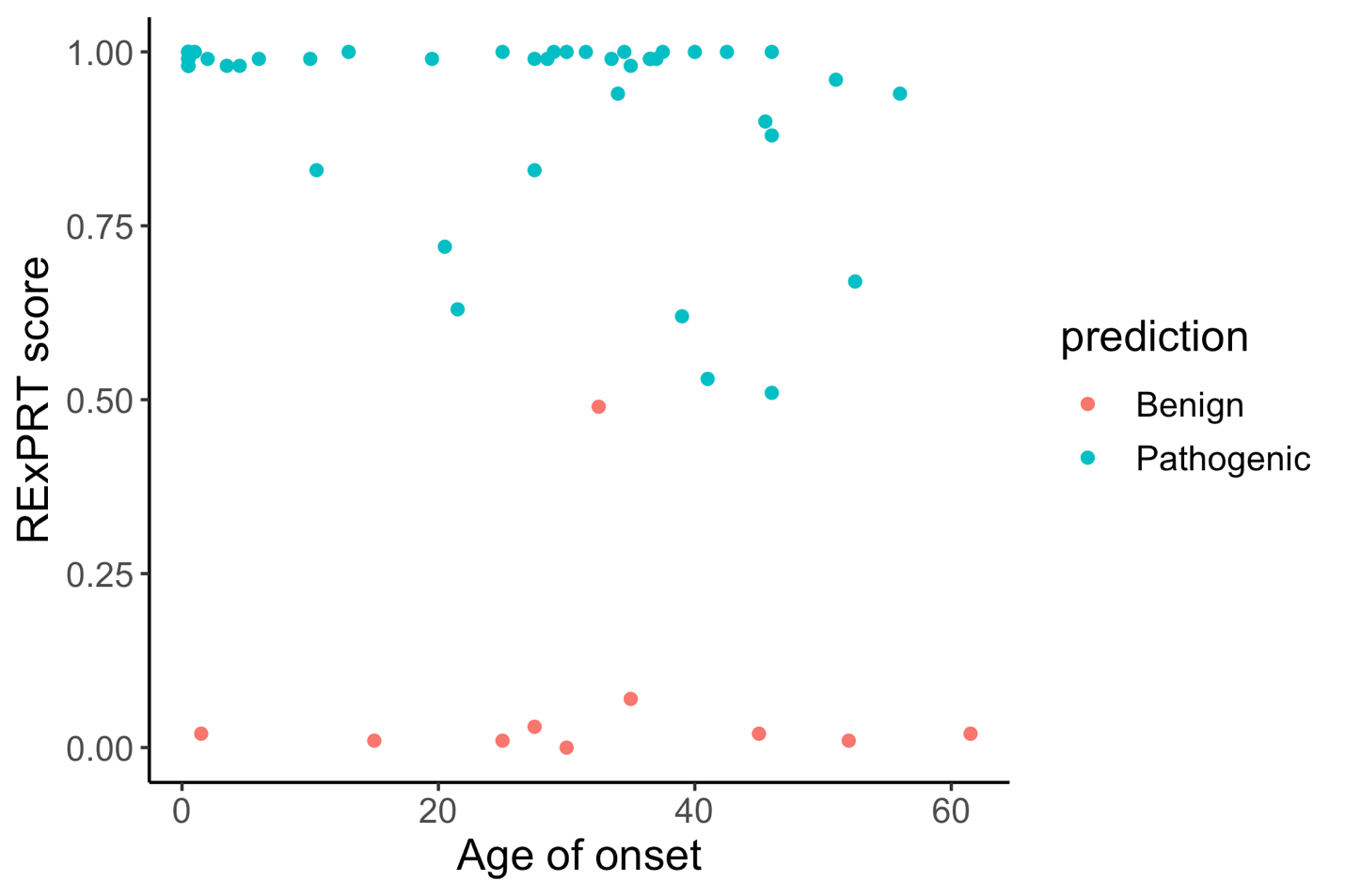


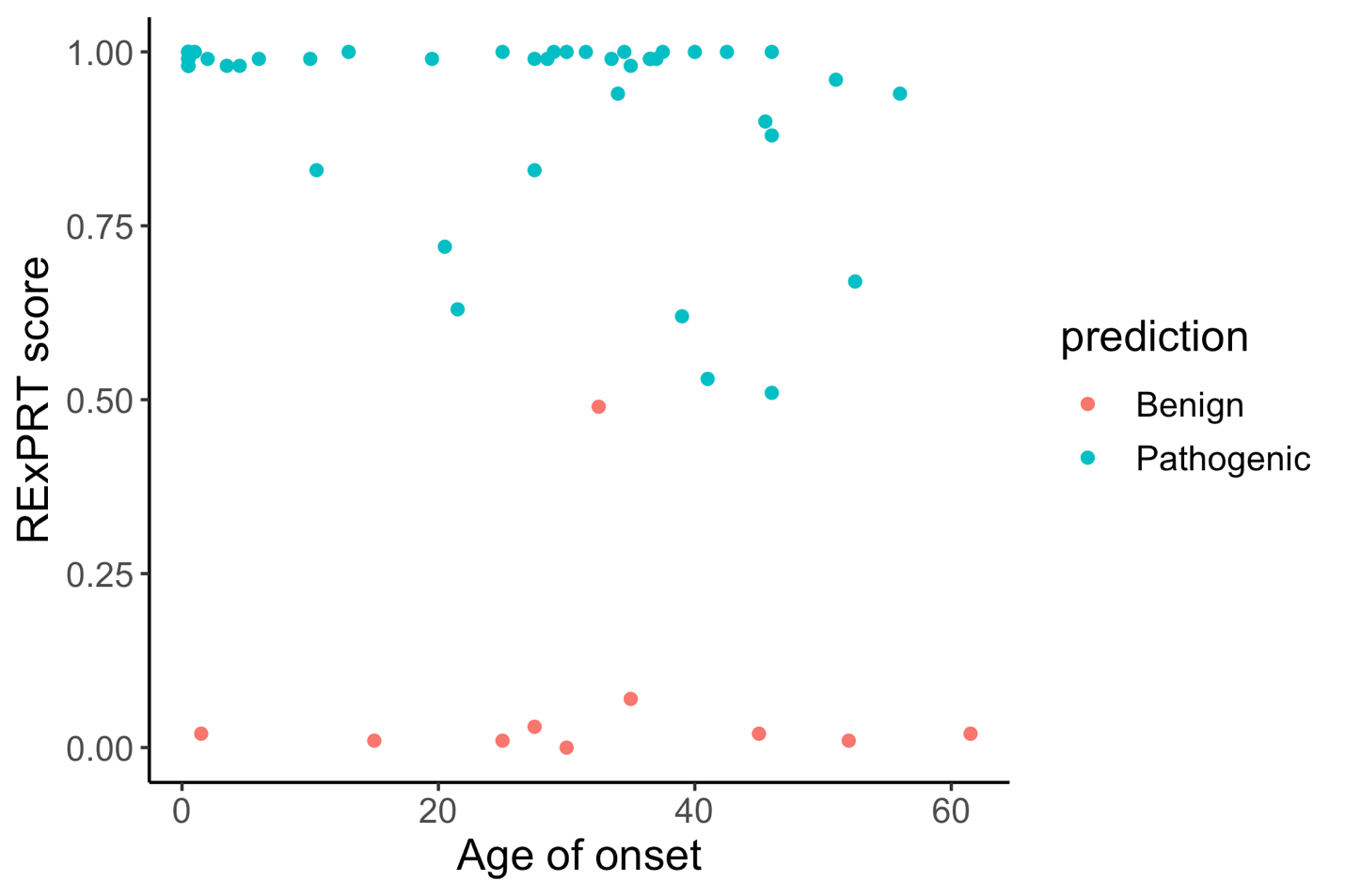


Predicted pathogenic

Predicted benign

**Fig. S2: Age of onset for known repeat expansion disorders and the maximum RExPRT pathogenicity score predicted for the associated TR locus.**

Age of onset is plotted as the midpoint between the range of onset ages observed according to the literature. The RExPRT score is plotted as the maximum between the SVM and XGB model predictions. RExPRT classifications of benign and pathogenic are color-coded as red and blue respectively. There is a weak negative correlation between age of onset and RExPRT score (spearman’s rho = -0.35), and many late onset disorders are correctly classified as pathogenic by RExPRT.
