# Supplementary material for: RExPRT: a machine learning tool to predict pathogenicity of tandem repeat loci
Source: Genome Biol. 2024 Jan 31;25:39. doi: 10.1186/s13059-024-03171-4 (PMC10832122; doi:10.1186/s13059-024-03171-4)
Supplement: Supplementary file 2 — Additional file 2. Review history. [file 13059_2024_3171_MOESM2_ESM.docx]

Review History

**First round of review**

**Reviewer 1**

**Were you able to assess all statistics in the manuscript, including the appropriateness of statistical tests used?** No.

**Were you able to directly test the methods?** No.

**Comments to author:**

The lack of prioritization tools to distinguish between pathogenic and benign TR expansions has posed a challenge in the field, despite TRs being implicated in 50 monogenic diseases. Existing tools mainly rely on constraint and pathogenicity correlations, which are effective for early onset diseases, but less applicable to late-onset diseases with minimal impact on natural selection. To address this, the authors developed a machine learning method called RExPRT. RExPRT utilizes an ensemble of two models (SVM and XGB) for superior performance. By applying RExPRT to the reference genome, approximately 30,000 pathogenic TRs were identified when expanded. Furthermore, when applied to UDN data, RExPRT pinpointed several candidate genes. While the concept of the paper is not groundbreaking and similar ideas have been used in SNP prediction, it fills a critical gap in the TR field. Both the method itself and its potential applications should be of interest to the field. Notably, certain features in the model, such as motif-related information from S2SNet, appear intriguing. The manuscript is well-organized and the statistical analyses are robust.

Regarding your specific questions:

1. Page 7,"we downloaded 30 different annotation datasets from a variety of sources including

UCSC's Table Browser, PsychENCODE, and relevant publications (Supplementary Table 1)":

a. Is there a special reason for that many brain/neuron related features are used, especially PsychENCODE? If there is tissue specificity or cell type specificity for the features used for training, the results may also bias to the specificity.

2. Page 7, "First, we created a list of 40 known pathogenic TRs which are listed as represented in the training dataset in Table 1":

a. Please include the diseases related to the TR in table 1, so that the readers know whether there may be some biases.

3. Page 7, "we used this reference TR dataset to create a set of "matched TRs", which included all TRs that have a disease motif and occur in the same genic region.":

a. Do the authors only use two features, i.e. the motif and genic location, to generate matched TRs? I am not sure what features may matter, but the length of the TR, the distance to the closest tss or the gene density in the local region should be considered in my opinion. Because this kind of features matters for sampling controls for SNPs.

b. I figured that the authors included a feature of the distance to the nearest gene in quantitative features. Because matched TRs were used for feature selection in the model, I think it's important to sample a good set of matched TRs using meaningful features.

4. Page 8, "Quantitative and multivalued features":

a. Please put these features in a table or ST1.

5. Page 16 and 17 , first two paragraphs of the results:

a. Please include the p values for all the places with OR, especially for "Upon further exploration, we found that there is significantly more enrichment of pathogenic TRs in the first exon of genes (OR = 113.60; 95% CI = [57.47, 223.43]) compared to the middle and last exons (Fig. 2c)."

b. For figure 2b and 2c, there is no matched TR overlapped with these elements?

6. Page 24, "fig 5":

a. Figure 5d doesn't have legend but figure 5f has. Please make it consistent.

7. Page 21, "RExPRT identifies ~30,000 TRs in the reference genome that may be pathogenic if expanded"

a. Does the results of fig 5f and fig 5g capture the same underlaying signals? From 5h and 5I, they seem different. Please explain and also include this explanation in the main text. What does the fraction stand for and what does the enrichment of fisher's exact test stand for.

8. How about the ability of the method to predict the pathogenicity TRs related to late-onset diseases? This question is initiated in the introduction but not answered then.

9. It seems that the current model performs well even with this imbalanced data. However, have the authors considered using specific methods designed for imbalanced data?

**Reviewer 2**

**Were you able to assess all statistics in the manuscript, including the appropriateness of statistical tests used?** No.

**Were you able to directly test the methods?** Yes.

**Comments to author:**

The authors described RExPRT (Repeat Expansion Pathogenicity pRediction Tool), which is designed to distinguish pathogenic from benign TR expansions. Leave-one-out cross validation results demonstrated that an ensemble approach comprised of support vector machines (SVM) and extreme gradient boosted decision tree (XGB) classified TRs with a precision of 92% and a recall of 90%. Application of RExPRT to ~800,000 TRs in the reference genome identified ~30,000 TRs that would be likely pathogenic upon expansion.

Although the machine learning approach to propose a tool for interpretation of the pathogenicity of expanded tandem repeats may be promising, there are concerns as described below.

1. Since the number of expanded TRs used for the machine learning is limited, there may be a strong bias and a limitation. In particular, the description of a precision of 92% and a recall of 90% is not convincing enough. In particular, there are no validation setts.

2. The following description is hard to understand for the reviewer. "Using bedtools fisher we ran Fisher's exact tests to provide an odds ratio, indicating if the amount of overlap between pairs of TR and annotation files is statistically significant given their coverage and the size of the genome." Clearer description including mathematical expressions is recommended.

3. Authors described "we used a set of 754 TR loci that were commonly expanded in the 1000 Genomes Project controls as negative training data.". The authors should provide the criteria for "common". How common are they?

4. Regarding the following description, "For analysis of Online Mendelian Inheritance in Man (OMIM) genes, we downloaded the list of genes from their website, and filtered for those that are Mendelian. For those genes that are dominant and recessive, we used this same dataset and filtered for the respective subtype.", the mode of inheritance may be different for diseases caused by expanded repeats.

5. The authors ran 2,982 genomes (968 probands and affected/unaffected family members) from the UDN through our outlier pipeline. Do the authors believe that the cases in the UDN have expanded repeats? Are they a good data set for testing RExPRT?

6. The authors described that 4,361 genes are associated with Mendelian diseases (OMIM). Is this correct? According to the latest information, 4,826 genes are listed as the total number of genes with phenotype-causing mutation.

7. The authors did not find any significant enrichment in introns. This is a major concern, since a substantial number of expanded TRs have been identified to be associated with disease, suggesting that data of pathogenic TRs derived from introns are not sufficient for machine learning.

8. The authors need to be careful in description of accuracy. Since the data are not sufficient for machine learning, it is too preliminary to describe the accuracy and sensitivity. The discussion seems to be a circular one. The notion is further supported by misclassification of four pathogenic TRs (RFC1, FXN, CNBP, and ATXN10). Description of the ROC curves is also preliminary.

9. Description of enrichment of pathogenic TRs in exons is not convincing as discussed by the authors as a limitation.

10. The focus of the manuscript is obscure. The authors tried to expand the description to the search for pathogenic TRs for neurological diseases. The authors should focus on development of RExPRT. Since there are many limitations as discussed above, direct application to the search for expanded TRs as the causative variants in neurological diseases is not fully warranted. The authors should discuss on various limitations of the current research.

11. It has been reported that some of the pathogenic expanded repeats are the results of insertion of new repeat motifs. How are these insertions evaluated?

**Authors Response**

**Point-by-point responses to the reviewers’ comments:**

**Reviewer #1:** The lack of prioritization tools to distinguish between pathogenic and benign TR expansions has posed a challenge in the field, despite TRs being implicated in 50 monogenic diseases. Existing tools mainly rely on constraint and pathogenicity correlations, which are effective for early onset diseases, but less applicable to late-onset diseases with minimal impact on natural selection. To address this, the authors developed a machine learning method called RExPRT. RExPRT utilizes an ensemble of two models (SVM and XGB) for superior performance. By applying RExPRT to the reference genome, approximately 30,000 pathogenic TRs were identified when expanded. Furthermore, when applied to UDN data, RExPRT pinpointed several candidate genes. While the concept of the paper is not groundbreaking and similar ideas have been used in SNP prediction, it fills a critical gap in the TR field. Both the method itself and its potential applications should be of interest to the field. Notably, certain features in the model, such as motif-related information from S2SNet, appear intriguing. The manuscript is well-organized, and the statistical analyses are robust.

Regarding your specific questions:

1. Page 7, "we downloaded 30 different annotation datasets from a variety of sources including UCSC's Table Browser, PsychENCODE, and relevant publications (Supplementary Table 1)":
   a. Is there a special reason for that many brain/neuron related features are used, especially PsychENCODE? If there is tissue specificity or cell type specificity for the features used for training, the results may also bias to the specificity.

- **Brain/neuron-related features:** Indeed, there is a specific rationale behind our choice to include a significant number of brain/neuron-related features in our analyses. The primary motivation stems from the fact that repeat expansion disorders predominantly manifest within the nervous system. Given this tissue specificity, we believed it was crucial to focus on features that are more likely to be relevant to these disorders, as opposed to selecting features from various cell types. Regarding our utilization of PsychENCODE specifically, we sought to obtain high quality Hi-C data from brain, which was readily accessible from this source. Our decision to leverage Hi-C data was inspired by methodologies used by Sun et al. who demonstrated enrichment of pathogenic repeat expansions at TAD boundaries.^1^
- **Bias:** It is important to acknowledge that by selecting features with brain/neuron specificity, we may potentially introduce bias towards identifying novel sites that follow similar patterns to those already known. However, we believe this bias is justifiable in the context of our study. Our supervised machine learning approach is designed precisely to leverage such biases. By training the model on features that are most relevant to repeat expansion disorders, we aim to improve its ability to recognize novel pathogenic TR expansions.

2. Page 7, "First, we created a list of 40 known pathogenic TRs which are listed as represented in the training dataset in Table 1":
   a. Please include the diseases related to the TR in table 1, so that the readers know whether there may be some biases.

- Thank you for the suggestion, we have now included disease names to Table 1.

3. Page 7, "we used this reference TR dataset to create a set of "matched TRs", which included all TRs that have a disease motif and occur in the same genic region.":
   a. Do the authors only use two features, i.e. the motif and genic location, to generate matched TRs? I am not sure what features may matter, but the length of the TR, the distance to the closest tss or the gene density in the local region should be considered in my opinion. Because this kind of features matters for sampling controls for SNPs.
   b. I figured that the authors included a feature of the distance to the nearest gene in quantitative features. Because matched TRs were used for feature selection in the model, I think it's important to sample a good set of matched TRs using meaningful features.

- Thank you for this inquiry. The generation of matched TRs was indeed based on the motif and genic location, in alignment with a methodology used by Sun at al., who found that disease-associated TRs are enriched in TAD boundaries. Here is why we focused on these specific criteria:
  i **Minimize bias**: while we acknowledge that other features, such as TR length, distance to the nearest TSS, or gene density in the local region, maybe relevant for generating matched TRs, we deliberately limited the selection criteria to the motif and genic location. The reason behind this approach is to avoid introducing potential biases. Factors like TR length and proximity to TSS can significantly impact TR pathogenicity and introducing such constraints might lead to biased sampling.
  ii **Robustness of the methodology**: Sun et al.’s study, which employed a similar methodology for generating matched TRs, provided robust and convincing results.^1^ By using this established approach, we aimed to ensure the robustness of our results while applying the methodology to identify additional relevant features beyond TAD boundaries.

4. Page 8, "Quantitative and multivalued features":
   a. Please put these features in a table or ST1.

- Thank you for the suggestion, we have now included these features in Supplementary Table 2.

5. Page 16 and 17, first two paragraphs of the results:
   a. Please include the p values for all the places with OR, especially for  "Upon further exploration, we found that there is significantly more enrichment of pathogenic TRs in the first exon of genes (OR = 113.60; 95% CI = [57.47, 223.43]) compared to the middle and last exons (Fig. 2c)."
   b. For figure 2b and 2c, there is no matched TR overlapped with these elements?

- **P-values**: Thank you for the suggestion, we have now included p-values for all reported ORs.
- **Matched TRs in figure 2**: Matched TRs were intentionally excluded from figures 2b and 2c, and we appreciate the reviewer’s keen observation. The rationale behind this exclusion is that matched TRs were specifically selected from different genomic regions. Consequently, they may already exhibit biases toward being enriched in certain genomic regions due to their nonrandom selection. The primary objective of figures 2b and 2c is to assess the enrichment of pathogenic TRs in specific genomic regions. To achieve this, it is more appropriate to compare pathogenic TRs to a broader reference group, such as simple repeats in general, rather than the matched TRs. Thank you for pointing out **this clarification, which we have now included in the manuscript**, and we hope this explanation addresses your concern. (*See page 16*)

6. Page 24, "fig 5":
   a. Figure 5d doesn't have legend but figure 5f has. Please make it consistent.

- Thank you for bringing this to our attention. To ensure consistency, we have made the following adjustment: the legend is now only included in the first instance where it is relevant in the muti-paneled figure. It is expected that readers understand that subsequent figures in the panel with the same color representation, follow the same legend.

7. Page 21, "RExPRT identifies ~30,000 TRs in the reference genome that may be pathogenic if expanded"
   a. Does the results of fig 5f and fig 5g capture the same underlaying signals? From 5h and 5I, they seem different. Please explain and also include this explanation in the main text. What does the fraction stand for and what does the enrichment of fisher's exact test stand for.

- All results presented in figure 5 aim to provide a comprehensive understanding of the enrichment of pathogenic TRs within specific genomic features. Each result is portrayed in two distinct plots:
  i Bar graph or pie chart: this represents the fraction of pathogenic or nonpathogenic TRs that overlap a particular feature relative to the total number of TRs within the group.
  ii Odds ratio graph: this graph evaluates whether the observed percentage above is significantly deviated from what would be expected by random chance, considering the coverage of that feature within the entire genome.
- When evaluating a feature, we employ bedtools fisher, which takes two sets of genomic intervals as input (e.g., pathogenic TRs and ORegAnno regions) and performs a Fisher’s exact test. This test is designed to determine whether there is a statistically significant nonrandom association between these two sets of genomic intervals. For the example provided, the presence of a significant p-value in the Fisher’s exact test indicates that the intervals in the pathogenic TRs are enriched in ORegAnno regions. It highlights that pathogenic TRs are more likely to occur in ORegAnno regions than expected by chance.
- You may observe scenarios where the percentage of pathogenic TRs overlapping one feature (e.g., ORegAnno) is higher than another feature (e.g., RAD21), but the odds ratio indicates greater enrichment within the RAD21 group. This discrepancy can be attributed to differences in the coverage of these features within the genome, as bedtools fisher considers this contextual information.
- **We have now included this explanation in the main text** to provide readers with a comprehensive understanding of the results and their interpretation. (*See pages 16, and 21-22*)

8. How about the ability of the method to predict the pathogenicity TRs related to late-onset diseases? This question is initiated in the introduction but not answered then.

- Thank you for raising this important point. We have taken your suggestion into consideration and conducted an analysis to assess the ability of RExPRT to predict pathogenicity of TRs associated with late-onset diseases. The results of this analysis have been included in Supplementary Figure 2, which presents a scatterplot showing the correlation between the age of onset for repeat expansion diseases, and the RExPRT pathogenicity score for the associated TR locus.
- Our analysis reveals a weak negative correlation between these two variables, with a Spearman’s rho value of -0.35. Notably, we observe that many late-onset disorders are correctly classified as pathogenic by RExPRT. **We believe this is an important finding, and it has been integrated into the main manuscript** to address the question you raised. (*See page 9*)

9. It seems that the current model performs well even with this imbalanced data. However, have the authors considered using specific methods designed for imbalanced data?

- Thank you for raising the issue of imbalanced data in our study.
  i **Metrics**: To account for this imbalance, we utilized metrics that are specifically designed for imbalanced datasets. These include the area under the Precision-Recall Curve (AUPRC) and the F1 score. These metrics are well suited for assessing model performance when dealing with imbalanced datasets.
  ii **Downsampling**: While downsampling is a common method for addressing imbalanced data, it was not a feasible option for our study. Our dataset and research objectives required us to retain all the TRs we were analyzing. Downsampling would have significantly reduced the already small dataset, potentially leading to the loss of important information and increasing the risk of overfitting.
  iii **Data augmentation**: While data augmentation practices could potentially be used in future studies, we opted not to artificially simulate loci to expand the training data set in our initial investigation. Our aim was to provide a foundation for understanding the pathogenicity of TRs without introducing artificial elements.
- In summary, we are conscious of the imbalanced nature of our data, and we have chosen metrics and approaches that are appropriate for addressing this challenge. We remain open to exploring additional techniques in future research and value your feedback. **This has now been added to the discussion.** (*See pages 14-15*)

**Reviewer #2:** The authors described RExPRT (Repeat Expansion Pathogenicity pRediction Tool), which is designed to distinguish pathogenic from benign TR expansions. Leave-one-out cross validation results demonstrated that an ensemble approach comprised of support vector machines (SVM) and extreme gradient boosted decision tree (XGB) classified TRs with a precision of 92% and a recall of 90%. Application of RExPRT to ~800,000 TRs in the reference genome identified ~30,000 TRs that would be likely pathogenic upon expansion.

Although the machine learning approach to propose a tool for interpretation of the pathogenicity of expanded tandem repeats may be promising, there are concerns as described below.

1. Since the number of expanded TRs used for the machine learning is limited, there may be a strong bias and a limitation. In particular, the description of a precision of 92% and a recall of 90% is not convincing enough. In particular, there are no validation setts.

- **Limited number of expanded TRs for machine learning**: we acknowledge that we are working in a data constrained environment and the limitation imposed by the relatively small number of true pathogenic TRs available for training. However, it’s important to note that it is deep learning methods that typically require substantially larger data set for optimal performance. Therefore, we opted for classical statistical learning techniques, which are known to perform well, even with smaller training datasets. These methods are robust and can still provide valuable insights from limited data. Furthermore, we employed leave-one-out cross-validation (LOOCV) specifically to mitigate the impact of the limited data set. LOOCV repeatedly trains the model on all but one data point, ensuring that each data point serves as a validation set exactly once. This approach allows for a more reliable estimate of model performance, even when limited by a small dataset.
- **Validation sets:** we understand the importance of validation sets in assessing model performance. In addition to LOOCV, which inherently serves us a form of cross validation, we incorporated a separate holdout test data of 10 pathogenic and 83 benign loci into our analysis. This test dataset has now been further updated by including 11 additional pathogenic loci, which enhances its representation and diversity.
- **Precision and recall values:** the precision of 92% and recall of 90% that we reported are from LOOCV on the training data. To provide a more comprehensive view of the model’s performance, we also included precision and recall values from the holdout test set. These updated values (94% precision and 76% recall) are comparable to those from LOOCV. We have provided a detailed explanation for the discrepancy in recall in the manuscript. (*See figure 4 and pages 7-8*)
- **Further evidence**: to further demonstrate the sufficiency of our data and the reliability of our results, we conducted additional analyses. We combined our current training, testing, and additional TR datasets, then generated five separate training and testing datasets by random division (2/3 for training and 1/3 for testing). We then employed the ensemble model and generated summary metrics for these datasets, which are now presented in Supplementary Table 3. These results are consistent with those presented in the original manuscript, further highlighting the robustness and high performance of our models across different circumstances. (*See pages 8-9*)

2. The following description is hard to understand for the reviewer. "Using bedtools fisher we ran Fisher's exact tests to provide an odds ratio, indicating if the amount of overlap between pairs of TR and annotation files is statistically significant given their coverage and the size of the genome." Clearer description including mathematical expressions is recommended.

- Thank you for bringing this to our attention. We have now rephrased this description in the manuscript. (*See page 16*)

3. Authors described "we used a set of 754 TR loci that were commonly expanded in the 1000 Genomes Project controls as negative training data.". The authors should provide the criteria for "common". How common are they?

- Thank you for highlighting the need for clarification in our manuscript regarding the criteria for selecting the 754 benign TR loci from the 1000 Genome Project controls. **We have now addressed this in methods section. (*See page 18*)**
- The 754 benign loci were chosen based on their prevalence in the control population. Specifically, we selected loci that were present at a size of >175 bp in more than 1% of the control samples. For more details, please refer to our manuscript published in *Scientific Data*.^2^

4. Regarding the following description, "For analysis of Online Mendelian Inheritance in Man (OMIM) genes, we downloaded the list of genes from their website, and filtered for those that are Mendelian. For those genes that are dominant and recessive, we used this same dataset and filtered for the respective subtype.", the mode of inheritance may be different for diseases caused by expanded repeats.

- Thank you for raising a valid point regarding the potential variability in modes of inheritance for diseases caused by repeat expansions. We recognize that the mode of inheritance can indeed differ, depending on whether a gene has a repeat expansion or other type of genetic variant (SNV, indel, etc.). For this reason, we chose to include all disease genes, regardless of their specific mode of inheritance, as our primary comparison group.
- We still believe that assessing the subgroups of genes (dominant and recessive) provides valuable insights. Notably, our findings suggest that pathogenic TRs are enriched in genes typically associated with dominant disorders. Nevertheless, it is important to acknowledge that most repeat expansion disorders are multifaceted in their mechanism of action, and often involve both gain and loss of function effects. **This has now been addressed in the discussion. (*See page 14*)**

5. The authors ran 2,982 genomes (968 probands and affected/unaffected family members) from the UDN through our outlier pipeline. Do the authors believe that the cases in the UDN have expanded repeats? Are they a good data set for testing RExPRT?

- We firmly believe that the UDN cohort is an excellent dataset for testing RExPRT for the following reasons:
  i. **Abundance of neurological phenotypes**: The UDN cohort exhibits a rich diversity of neurological and musculoskeletal phenotypes, which aligns well with the typical manifestations of repeat expansion disorders. These disorders are known to primarily affect the nervous system and often result in complex clinical presentations, mirroring the complexity observed in UDN cases.
  ii. **Likelihood of complex genetic variants**: The UDN cohort comprises individuals who have been challenging to genetically diagnose through conventional means. These undiagnosed cases are likely to encompass a range of more complex genetic variants, including structural variants and repeat expansions.
  iii. **Diagnosis of known repeat expansion disorders:** in another ongoing study that we are preparing for publication, we employed a genotype-first approach to diagnose nine patients within the UDN cohort with known repeat expansion disorders (but unusual symptom presentations). Additionally, we identified six patients with expansions in known disease genes, pending further confirmation. These findings underscore the potential for discovering novel pathogenic repeat expansion genes in the UDN cohort.

6. The authors described that 4,361 genes are associated with Mendelian diseases (OMIM). Is this correct? According to the latest information, 4,826 genes are listed as the total number of genes with phenotype-causing mutation.

- Thank you for pointing out the updated information regarding the number of genes associated with Mendelian diseases in OMIM. Since the time of writing this manuscript, it is evident that new genes have been added. It’s important to note that the figure we provided in the manuscript only includes genes associated with Mendelian disorders, as repeat expansions typically fall under this category. We have now updated the total number of genes to reflect the latest information. **We appreciate your diligence in ensuring the accuracy of our work. (*See page 3*)**

7. The authors did not find any significant enrichment in introns. This is a major concern, since a substantial number of expanded TRs have been identified to be associated with disease, suggesting that data of pathogenic TRs derived from introns are not sufficient for machine learning.

- We agree with the reviewer about the substantial number of known pathogenic intronic TRs and felt this observation warranted careful inspection. Fisher’s exact test was employed to assess whether the representation of intronic loci within the known pathogenic TRs used for training significantly deviates from what would be expected by chance. Our analysis reveals that pathogenic TRs are actually not enriched in introns; even though we all agree that several known pathogenic TRs are located in introns, specifically in first introns. This merely serves as a reminder of the incredibly large total number of intronic TRs in the human genome. We thus should not expect differently from ReXPRT, which shows the absence of significant enrichment of pathogenic TRs in introns.
- Several factors may contribute to this observation. Firstly, intronic loci, have historically been more challenging to study and validate compared to exonic regions, potentially leading to their underrepresentation in the known pathogenic TR dataset. Secondly, it is plausible that intronic loci are less prone to pathogenicity even when expanded since these regions tend to be less genetically constrained than exonic regions. Nevertheless, this **lack of enrichment in introns does not imply that intronic TRs should be excluded from machine learning approaches.**
- We would like to emphasize that our machine learning model incorporates multiple features beyond the intronic/exonic categorization to assess the pathogenicity of TRs. These additional features are critical in providing a comprehensive evaluation of TRs associated with diseases. In essence, our approach considers as a broader spectrum of information, ensuring a more accurate assessment of TR pathogenicity, regardless of their genomic location.

8. The authors need to be careful in description of accuracy. Since the data are not sufficient for machine learning, it is too preliminary to describe the accuracy and sensitivity. The discussion seems to be a circular one. The notion is further supported by misclassification of four pathogenic TRs (RFC1, FXN, CNBP, and ATXN10). Description of the ROC curves is also preliminary.

- We respectfully disagree with the assertion that our data are insufficient for machine learning. We believe that our approach is robust and well-suited for several reasons:

i. **Statistical learning approach**: it’s important to note that we utilized classical statistical learning methods rather than deep learning, which typically requires larger data sets. The use of support vector machines (SVM) is known to be powerful even with smaller data sets.^3^
ii. **Biological datasets**: healthcare-related data, particularly in the context of genetics and rare diseases are often limited in size due to the nature of the data and ethical considerations.^4^ While the dataset is relatively small, it does not render machine learning approaches inappropriate. Several published studies have successfully employed machine learning with smaller data sets in healthcare:
    a. Walse et al. used 400 instances for a classification model in chronic kidney disease.^5^
    b. Lysdahlgaard S. trained a model using 131 CT scans of the liver.^6^
    c. Ko et al. developed a machine learning approach for predicting cancer outcomes with a small dataset.^7^
iii. **p < n:** in our case, the number of features (p) is smaller than the number of TRs (n). This feature-to-instance ratio is favorable for our analysis.
iv. **Robust results**: the inclusion of a test dataset demonstrates that our models continue to perform well, mitigating concerns of potential overfitting, which is often associated with smaller datasets. For comprehensiveness, we have provided a range of metrics, including accuracy, precision, recall, and F1 score to assess the performance of our model. Our approach has shown its reliability, particularly in terms of precision.
v. **Enrichment in disease genes**: in figure 5i we present compelling evidence that RExPRT-predicted pathogenic TRs are enriched in disease genes. Notably, this was not a feature explicitly trained in our model. This finding underscores the capability of RExPRT to autonomously learn and identify biologically relevant associations, further strengthening the significance of our results.

- **Misclassification**: misclassifications are an inherent part of any classification model. While we acknowledge the misclassification of four pathogenic TRs in our training dataset, it is important to emphasize that no method, even in larger datasets, is entirely free from misclassifications and that a model does not need to have perfect performance on its training data in order to have learned real patterns from that data or to be useful in practice.
- **Further evidence**: to further demonstrate the sufficiency of our data, we conducted additional analyses. We combined our current training, testing, and additional TR datasets, then generated five separate training and testing datasets by random division (2/3 for training and 1/3 for testing). We then employed the ensemble model and generated summary metrics for these datasets, which are now presented in Supplementary Table 3. These results are consistent with those presented in the original manuscript, further highlighting the robustness and performance of our models across different circumstances. This additional evidence reinforces the reliability of our findings and suitability of our dataset for machine learning analysis. (*See pages 8-9*)

9. Description of enrichment of pathogenic TRs in exons is not convincing as discussed by the authors as a limitation.

- While we obviously acknowledge the limitations we described in the discussion, we maintain that this association is meaningful. We have supported our statements regarding enrichment of pathogenic TRs in exons with robust statistical analyses. The observed odds ratio for known pathogenic TRs in exons is 86, with a highly significant p-value <2.2e^-16^. RExPRT-classified pathogenic TRs exhibit an odds ratio of 53 with a similarly low p-value. In interpreting this result, it is important to note that we are using a supervised machine learning approach here. Therefore, RExPRT will identify novel pathogenic TRs that follow similar patterns to those that it was trained on. **This point has now been added to the discussion.** (*See page 13*)

10. The focus of the manuscript is obscure. The authors tried to expand the description to the search for pathogenic TRs for neurological diseases. The authors should focus on development of RExPRT. Since there are many limitations as discussed above, direct application to the search for expanded TRs as the causative variants in neurological diseases is not fully warranted. The authors should discuss on various limitations of the current research.

- **Obscure focus**: Our primary objective in the study was to develop and introduce the RExPRT method. Secondarily, we illustrated RExPRT’s application to the search for pathogenic TRs in neurological diseases, a practice that aligns with similar approaches in other research studies. For instance, the development of PrimateAI-3D, as described in *Science*, also involved the application of their machine learning variant interpretation tool to identify candidate genes in four different patient cohorts.^8^ In the manuscript introducing AlphaMissense in *Science*, the authors also provided a resource of variant effect predictions for all possible missense variants.^9^ Similarly, we generated a list of TRs of unknown significance and used RExPRT to assess their pathogenicity.
- **Validation**: The core purpose of RExPRT is to identify and present strong candidate TRs that can serve as a starting point for further functional studies. To support this, we conducted preliminary validation of the FAM193B locus, classified as pathogenic by RExPRT in the UDN cohort. We deemed functional validation of this locus to be outside the scope of the current manuscript but have conducted that work for a future manuscript. We observed significantly increased transcript levels in patients compared to controls, which provides an initial indication of functional validation, and highlights the potential of RExPRT in selecting promising candidates.
- **Limitations**: Like any research endeavor, we recognize the importance of openly discussing the limitations of our work. **We have a dedicated section in the discussion to address these limitations and have expanded on them in response to your feedback. (*See pages 13-15*)**

11. It has been reported that some of the pathogenic expanded repeats are the results of insertion of new repeat motifs. How are these insertions evaluated?

- Thank you for this insightful question. RExPRT operates without knowledge of the origin of the repeat, whether it results from the expansion of a reference repeat, change in the reference motif, or was inserted de novo. While RExPRT is not specifically designed to detect de novo repeat insertions from WGS data, if provided with the coordinates of a de novo inserted TR, it can still classify the TR and assess its pathogenicity. In summary, RExPRT’s ability to evaluate TRs extends to de novo insertions and motif changes when the relevant information is provided.

**References**

1	Sun, J. H. *et al.* Disease-Associated Short Tandem Repeats Co-localize with Chromatin Domain Boundaries. *Cell* **175**, 224-238 (2018).

2	Fazal, S. *et al.* Large scale in silico characterization of repeat expansion variation in human genomes. *Scientific Data* **7**, 294 (2020).

3	Kramer, K. A., Hall, L. O., Goldgof, D. B., Remsen, A. & Luo, T. Fast Support Vector Machines for Continuous Data. *IEEE Transactions on Systems, Man, and Cybernetics* **39**, 989-1001 (2009).

4	Banerjee, J. *et al.* Machine learning in rare disease. *Nature Methods* **20**, 803–814 (2023).

5	Walse, R. S. *et al.* Effective Use of Naïve Bayes, Decision Tree, and Random Forest Techniques for Analysis of Chronic Kidney Disease. *Smart Innovation, Systems and Technologies*, 237-245 (2020).

6	Lysdahlgaard, S. Comparing Radiomics features of tumour and healthy liver tissue in a limited CT dataset: A machine learning study. *Radiography* **28**, 718-724 (2022).

7	Ko, S., Choi, J. & Ahn, J. GVES: machine learning model for identification of prognostic genes with a small dataset. *Scientific Reports* **11**, 439 (2021).

8	Gao, H. *et al.* The landscape of tolerated genetic variation in humans and primates. *Science* **380**, 929 (2023).

9	Cheng, J. *et al.* Accurate proteome-wide missense variant effect prediction with AlphaMissense. *Science* **381**, 1303 (2023).

**Second round of review**

**Reviewer 1**

My questions are well addressed. Only one more question is raised because authors added another analysis. Please check bellow.

8. How about the ability of the method to predict the pathogenicity TRs related to late-onset

diseases? This question is initiated in the introduction but not answered then.

 Thank you for raising this important point. We have taken your suggestion into

consideration and conducted an analysis to assess the ability of RExPRT to predict

pathogenicity of TRs associated with late-onset diseases. The results of this analysis have

been included in Supplementary Figure 2, which presents a scatterplot showing the

correlation between the age of onset for repeat expansion diseases, and the RExPRT

pathogenicity score for the associated TR locus.

 Our analysis reveals a weak negative correlation between these two variables, with a

Spearman’s rho value of -0.35. Notably, we observe that many late-onset disorders are

correctly classified as pathogenic by RExPRT. We believe this is an important finding, and

it has been

New question: There is no method related to this analysis. Please have a check. The last claim "

Notably, we observe that many late-onset disorders are correctly classified as pathogenic by RExPRT" has no evidence attached. Add a ST or something else please.

**Reviewer 2**

According to Walley et al. BMC Health Services Research (2018) 18:652 https://doi.org/10.1186/s12913-018-3458-2, the mean age at symptom onset (years, mean ± SD) is 11.11 ± 18.0, raising a possibility that patients with onset in adulthood may not be well recruited to UDN. Since there are many patients with late adulthood onset who carry expanded repeats, the authors need to mention this point as a limitation.

**Authors Response**

**Point-by-point responses to the reviewers’ comments:**

Reviewer #1: My questions are well addressed. Only one more question is raised because authors added another analysis. Please check bellow.

8. How about the ability of the method to predict the pathogenicity TRs related to late-onset diseases? This question is initiated in the introduction but not answered then.

- Thank you for raising this important point. We have taken your suggestion into
  consideration and conducted an analysis to assess the ability of RExPRT to predict
  pathogenicity of TRs associated with late-onset diseases. The results of this analysis have been included in Supplementary Figure 2, which presents a scatterplot showing the correlation between the age of onset for repeat expansion diseases, and the RExPRT pathogenicity score for the associated TR locus.
- Our analysis reveals a weak negative correlation between these two variables, with a
  Spearman’s rho value of -0.35. Notably, we observe that many late-onset disorders are correctly classified as pathogenic by RExPRT. We believe this is an important finding, and it has been integrated into the main manuscript to address the question you raised. (*See page 9*)

New question: There is no method related to this analysis. Please have a check. The last claim "Notably, we observe that many late-onset disorders are correctly classified as pathogenic by RExPRT" has no evidence attached. Add a ST or something else please.

- Thank you for your feedback. In response to your suggestion, we have added details of the method used for the analysis in the Methods section of the manuscript. Specifically, we calculated the midpoint of the observed age of onset ranges for each disorder. Next, we generated a scatter plot where each point corresponds to a disease-associated pathogenic TR locus. The x-axis represents the midpoint of the observed range of onset ages for the associated disease (refer to Table 1), while the y-axis reflects the RExPRT ensemble score. Subsequently, we calculated the Spearman’s rank correlation coefficient (rho) for these two variables.
- In Figure S2, we present the scatter plot illustrating the correlation between the age of onset for repeat expansion disorders and the RExPRT pathogenicity score for the associated locus. Notably, the blue points on the scatter plot denote TR loci that RExPRT correctly classified as pathogenic. To support our claim, we observe that, considering the midpoint of age ranges observed (as plotted in Figure S2), ten out of 13 diseases with onset after age 40 were classified as pathogenic. It is important to note that repeat expansion disorders tend to have wide variability in their ages of onset.

Reviewer #2: According to Walley et al. BMC Health Services Research (2018) 18:652 (<https://bmchealthservres.biomedcentral.com/articles/10.1186/s12913-018-3458>), the mean age at symptom onset (years, mean ± SD) is 11.11 ± 18.0, raising a possibility that patients with onset in adulthood may not be well recruited to UDN. Since there are many patients with late adulthood onset who carry expanded repeats, the authors need to mention this point as a limitation.

- Thank you for bringing up this important point, we acknowledge the limitation regarding late onset disorders not being well represented in the UDN cohort. It is noteworthy that the UDN focuses on recruiting complex and severe cases, and as a result we have encountered instances of juvenile onset for disorders that are typically associated with later onset, such as Huntington’s disease. We also recognize that many repeat expansion disorders exhibit a broad range of symptom onset. We appreciate your valuable feedback and have now addressed this in the manuscript.
